# Fluorescence-based assay as a new screening tool for toxic chemicals

**DOI:** 10.1038/srep33922

**Published:** 2016-09-22

**Authors:** Ewa Moczko, Evgeny M. Mirkes, César Cáceres, Alexander N. Gorban, Sergey Piletsky

**Affiliations:** 1 Universidad Católica de la Santísima Concepción, Facultad de Ciencias, Departamento de Química Ambiental, Alonso de Ribera 2850, Concepción, Chile; 2 Department of Mathematics, University of Leicester, UK; 3 Department of Analytical and Inorganic Chemistry, University of Concepción, Chile; 4 Department of Chemistry, University of Leicester, UK

## Abstract

Our study involves development of fluorescent cell-based diagnostic assay as a new approach in high-throughput screening method. This highly sensitive optical assay operates similarly to e-noses and e-tongues which combine semi-specific sensors and multivariate data analysis for monitoring biochemical processes. The optical assay consists of a mixture of environmental-sensitive fluorescent dyes and human skin cells that generate fluorescence spectra patterns distinctive for particular physico-chemical and physiological conditions. Using chemometric techniques the optical signal is processed providing qualitative information about analytical characteristics of the samples. This integrated approach has been successfully applied (with sensitivity of 93% and specificity of 97%) in assessing whether particular chemical agents are irritating or not for human skin. It has several advantages compared with traditional biochemical or biological assays and can impact the new way of high-throughput screening and understanding cell activity. It also can provide reliable and reproducible method for assessing a risk of exposing people to different harmful substances, identification active compounds in toxicity screening and safety assessment of drugs, cosmetic or their specific ingredients.

In recent years scientists are facing a growing pressure to move from conventional approaches evaluating toxic potential of the products and safety assessment of chemical compounds towards modern, inexpensive and more efficient *in vitro* methods^1^,^2^,^3^. A present surge in new initiatives not only among industrial scientists around the world but also academic researchers, promotes high-throughput *in vitro* methods and more human-relevant, non-animal systems^4^,^5^,^6^. Novel approaches introduce the research concept of the reduction, refinement, or even obviation the need for animals in research studies and toxicity testing. Majority of these techniques include cell-based (*in vitro*) methods such as stem cell technologies, tissue engineering, organs-on-chips^7^,^8^,^9^,^10^,^11^,^12^ or ‘omics technologies (including genomics, epigenomics, and proteomics)^13^,^14^,^15^. Recently, rapidly developing are computational (*in silico*) toxicology and modeling techniques which have already revealed moderate success in advancing toxicity testing and risk assessment. They also promise enhancement and efficiency (time and price to solution) of the existing methods^16^,^17^. Despite the advantages, they still have to be coupled with experiments to prove adequacy of theoretical models which are applied to make predictions. Among variety of approaches, strategies and types of possible screening assays in toxicity evaluation the ones which have attracted increasing interests are *in vitro* cell-based sensing methods applying fluorescence^18^,^19^,^20^. They have demonstrated number of advantages in comparison to other, non-fluorescence methods such as higher sensitivity and the flexibility of using multi-wavelength option for simultaneously detection of the emission of different fluorophores, the decay time or the polarization of the fluorescence emission^21^,^22^,^23^,^24^. In toxicology studies, the use of combined fluorescent *in vitro* assays and *in silico* methods has a great potential to identify toxicants faster and easier, reducing the need for expensive complicated high-throughput screening techniques and whole-animal models^14^,^15^,^16^. Herein, we present a new concept for toxicity assays and data evaluation. Our approach combines cell-based optical method with multivariate data analysis as a novel, promising scientific strategy for assessing the safety of chemicals. A schematic illustration of the presented concept is shown in Fig. 1. The operating principle of the assay is similar to electronic noses and tongues systems which mimic mammalian smell and taste recognition, and their optical analogue previously used by authors for quantitative and qualitative analysis of the samples^25^,^26^. In particular, optical dyes array has been developed and optimized for simultaneous quantitative measurements of several physicochemical parameters, monitoring of growing cell cultures and identification of gastrointestinal diseases in humans.

In recent studies, we improved the assay and enhanced its performance by applying computerized cell-based fluorescence system. The novelty of this work lies in a combination of dyes with human skin cells where cells produce characteristic response to toxic chemicals. Different toxicity effects of specific compounds are reported by dyes and reflected in the changes of their fluorescence spectra. For *in vitro* studies we have used human skin cells. For mathematical feature extraction we employed the dimensionality reduction methods: transformation of the set of fluorescent images into their cross-correlation space and principal component analysis (PCA)^27^ in this space. The classical heuristics (the Kaiser rule^28^ and the broken stick model^29^) advise to retain 4 or 5 principal components. To distinguish active from non-active chemicals they were used classification algorithms in the space of five first principal components: Fisher’s discriminant^30^, logistic regression^31^, kNN^32^, advanced kNN^33^, decision trees^34^ and various probability density function estimators^35^,^36^.

Already Fisher’s linear discriminant gave good result for toxicity diagnosis with specificity of 91% and sensitivity of 86% in Leave-One-Out-Cross-Validation (LOOCV) test and 90%-89% on the randomly selected 19-element test set. Advanced 3NN classification gave specificity of 97% and sensitivity of 93% in LOOCV test and specificity of 100% with sensitivity of 89% on the test set. Other nonlinear methods gave similar results. (Specificity represents the number of true negatives, in particular specificity of 97% means that 97 out of 100 non-toxic chemicals are correctly classified, and sensitivity represents the number of true positives, thus sensitivity of 93% means that 93 out of 100 toxic chemicals are correctly classified). Very promising results proved that this technique offers possible alternative to the improvement or even replacement of exiting methods which enable identification of various analytes and safety assessment whether a drug, cosmetic or their specific ingredients are going to be harmful to humans, other animals or environment. Additionally, such non-animal method would be cheaper, quicker and more effective compare to long term animal testing.

## Results

### Characterization of the optical toxicity assay

**Figure 2:**
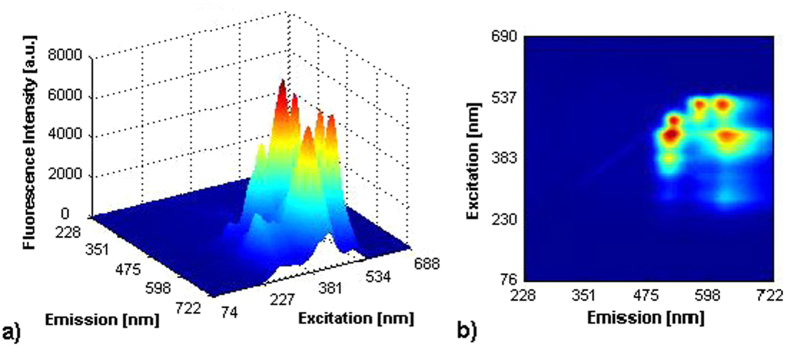
Three-dimensional color mapped surface diagram (**a**) and color filled contour diagram (**b**) of 5 fluorescent dyes: 8-Hydroxypyrene-1′,3,6-Trisulfonic Acid, Oregon Green 514, is Rhodamine B, Tris(4,7-diphenyl-1,10-phenanthroline) ruthenium dichloride, Thionin Acetate. Measurements were performed in 50 mM PBS buffer at pH 7.4.

Our fluorescent-based assay relies on operating principle of e-noses and e-tongues and it has been applied already for quantitative and qualitative analysis of complex biological and clinical samples^25^,^26^. The fluorescent dyes were selected in the way that their mixture allows discreet reading of emission maxima with the wavelengths separated by at least 20 nm. For the minimum interference with biological samples they were responsive in the VIS-NIR range. In present work selected dyes were applied to monitor responses of human skin cells in the presence of chemical agents with different toxic properties, which may cause damage to human skin. In current studies (Methods) we cultivated human skin cells and incubated them with different chemicals which triggered specific cellular responses. The mixture of five fluorescent dyes added to the solutions generated distinctive fluorescence spectra characteristic for toxic properties of tested chemicals. Control measurements were performed using samples without growing cells. The sample of fluorescence spectra obtained is presented in Fig. 2. Using chemometric approaches patterns of assay responses were further analyzed and the optical signals were related to the analytical characteristics of the samples.

### Data analysis

For each chemical we obtained two digitized fluorescence images: with growing cells and without growing cells (control). Both images are two-dimensional 511 × 511 arrays of fluorescence intensities as functions of emission and excitation (Fig. 2). We tested 34 irritating (IRR) and 28 non-irritating (Non-IRR) compounds (62 chemicals in total). Tree classification problems were solved:

1. Predict the class of compound (IRR or Non-IRR) using the control fluorescence image (without growing cells) as the input;
2. Predict the class of compound using the fluorescence image with growing cells as the input;
3. Predict the class of compound using both input images (with and without cells).

**Figure 3:**
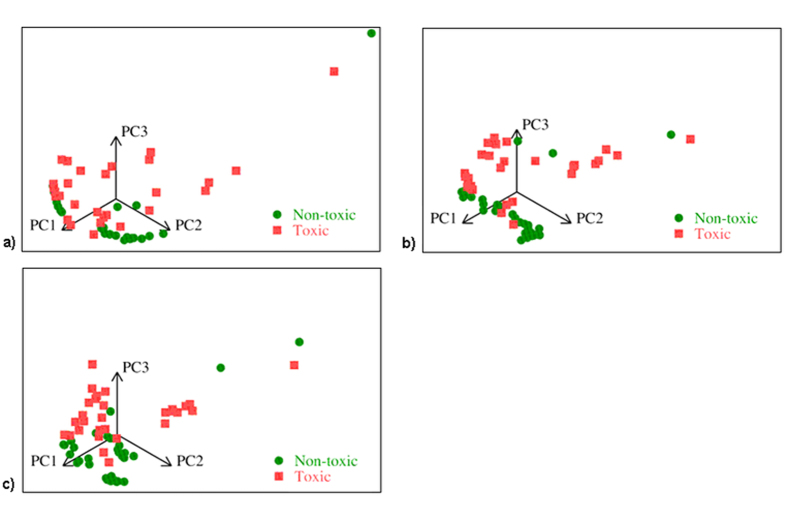
Samples in the space of the first three principal components: (**a**) samples with the control fluorescence images as the inputs (for the classification problem 1), (**b**) samples with the fluorescence image with growing cells as the inputs, (for the classification problem 2), (**c**) samples with both input images (with and without cells) as inputs (for the classification problem 3). Visually, class compactness and separability of classes increases from (**a**) to (**c**).

For the first and the second classification problems the dimension of input is 511 × 511 = 261,121 and for the third problem it is 2 × 511 × 511 = 522,242. These dimensions are much bigger than the number of samples available (62). Therefore, the first preprocessing task was dimensionality reduction. We transformed the set of fluorescent images into cross-correlation space and apply principal component analysis in this space. Then we select the principal components for retaining using the Kaiser rule or the broken stick model. The developed approach reduced multidimensional input space of fluorescent images to four or five features. We retained five principal components in the cross-correlation space. Data in the space of the first three principal components are presented in Fig. 3a–c for different sets of inputs.

Separability and compactness of classes increase from the case a (control, compounds without cells) to the case c (inputs include both the control fluorescent image and the image for compounds with growing cells).

The first classification algorithm we applied is the simple and robust Fisher discriminant. Its classification results of the whole set of samples are presented in Table 1. It was not surprising that for the task 1 (control, without cells) the quality of classification was not satisfactory but for data of fluorescence with cells the linear discriminant analysis in the five-dimensional feature space worked quite well and its performance was further improved slightly for the combined inputs (task 3).

We tested several families of nonlinear classifiers to improve the classification performance: K nearest neighbors (KNN), decision tree (DT), Gaussian mixture (GM), radial-basis functions for probability density function estimation (PDFE), weighted logistic regression (LR), naïve Bayes classifier (NB), Fisher’s linear discriminant analysis (LDA). Detailed description of the methods and their use was presented in the biomedical case study^37^.

Results of the best methods in the listed families are collected in Table 2 (for input fluorescence images with growing cells) and Table 3 (for input fluorescence images with and without cells together). The number of samples for testing is indicated in parentheses. Thus, LOOCV (61+1) means that we used all 62 samples for future extraction, selected one sample for testing, 61 samples for training and repeat this operation in a loop for every such splitting. Training set test (43) and test set test (19) mean that we split the samples into a test set with 19 samples and a training set with 43 samples, used these 43 samples to extract features, trained and tested the classification algorithms on the training set and then test them on the test set which is not seen before. The results of all methods in all three tests (LOOCV 61+1, test set test, and training test set) were quite good. There was no sign of overfitting because the difference between the training and the test sets tests was not large. The performance of Fisher’s linear discriminant in the tests was not significantly worse than the performance of the nonlinear methods. The 3NN method worked uniformly well and at this stage it can be recommended. It is the KNN method with *k* = 3, the adaptive distance, and the triangular kernel for distance transformation and voting. The optimal weight of Non-IRR class was 0.45.

## Discussion

Human skin cells in the presence of fluorescent dyes interacted with different chemical agents which triggered cellular response, producing distinctive fluorescence patterns characteristic for its toxic properties. The optical signals were further processed and transformed into qualitative information whether compounds were toxic or not. To distinguish active from non-active chemicals, we employed dimensionality reduction and tested several families of classification algorithms in the five-dimensional space of extracted features: Fisher’s discriminant, logistic regression, kNN, advanced kNN, decision trees and various probability density function estimators. The best methods from these families gave both specificity and sensitivity above 80%, and specificity + sensitivity above 170%, where specificity represented the number of true negatives and sensitivity represented the number of true positives. Good performance of all tested families of the classification methods indicated that the physicochemical method developed allowed us to distinguish between toxic (IRR) and non-toxic (Non-IRR) compounds with confidence. The 3NN method worked uniformly well and demonstrated in LOOCV testing sensitivity of 97% and specificity of 93% (for inputs with and without cells). At this stage it can be recommended for further use with these combined inputs.

Our results demonstrated that the optical assay can be successfully integrated with cell cultures and the physicochemical changes produced by cells in the contact with different compounds reflected in the changes of fluorescent pattern of dyes. This approach has several important advantages compared with traditional biochemical assays, such as lower cost, short time of measurements, high sensitivity and reproducibility. The major benefits of this technique also include possibility to develop automatic system to screen large number of samples quickly and efficiently, and to decipher molecular mechanism of toxic action. It can be employed in high-throughput screening, in particular in safety assessment of drugs, cosmetics, specific ingredients and provide alternative screening tool in medical research, pharmaceutical industry and other areas of chemical testing.

## Methods

### Composition of dye assay

The assay was composed of five commercially available fluorescent dyes (see Table 4). For the minimum interferences with biological samples, selected fluorescent dyes were responsive in the VIS-NIR range. They have already been successfully applied as analytical tool for qualitative measurements of several physicochemical parameters such as pH, temperature, dissolved oxygen or ionic strength of a solution^25^ and in biomedical diagnostics^26^. The mixture of fluorescent dyes were prepared as a stock solution of following dyes: 0.15 mM 8-Hydroxypyrene-1′,3,6-trisulfonic acid, 0.1 mM Rhodamine B, 2 mM Thionin acetate, 0.025 mM Oregon Green 514, 6 mM Tris (4,7 – diphenyl - 1,10 - phenanthroline) ruthenium dichloride. The solutions were prepared in deionised water and stored at ~5 °C, covered with aluminium foil to protect them from light. The 200 μl of each dye stock solutions were mixed together and used in further experiments.

### Samples preparation

The cell line used in the experiments was human keratinocytes (HaCaT) purchased from American Tissue Culture Collections (ATCC, Manassas, VA). DMEM (Dulbecco’s Modified Eagle’s medium) with L-glutamine and high glucose was purchased from Life Technologies (Invitrogen), UK. Trypan Blue, TrypLE and FBS (Fetal Bovine Serum), animal origin free were also purchased from Life Technologies (Invitrogen). Penicillin (P), streptomycin (S) and all chemicals used for testing of the optical assay were purchased from Sigma-Aldrich. Cells were grown at 37 °C in T75 cm^2^ tissue culture flask in DMEM medium with 10% FCS and 1% P/S for three days to reach at least 80% of confluency. Then the cells were splitted by removing old medium, rinsing them with PBS (phosphate buffer saline) and detaching them from the wells of the flask using 2–3 mL of TrypLE. Cells with reagent were left in 37 °C/5% CO_2_ incubator for about 10 min. After all cells were detached, 3 mL of DMEM containing serum was added to the flask to inactivate TrypLE. Before the cells were resuspended in fresh medium, they density and viability was checked using automatic cell counter and Trypan Blue staining to calculate a split ratio required to obtained the right, constant number of cells for experiments and subcultering. All experiments were performed at cells density of 1 × 10^5^ cells/ml. The chemicals in toxicity tests were used at the concentrations of 0.1 mM for ones which cause irritation and 0.1 μm or less of the safe ones which should not cause any harm to human skin. All experiments were performed in triplicate. Applying the mixture of fluorescent dyes into the suspensions of cells did not change significantly their viability compared with toxic compounds and therefore was neglected.

### Instrumentation

The measurements of fluorescence intensity have been performed using three-dimensional spectrofluorimeter Jobin Yvon – SPEX FL-3D (Instruments SA, Stanmore, Middlesex, UK) at 0.5 s of time exposure. The spectra have been recorded over a range of excitation (74–691 nm) and emission (227–724 nm) wavelengths. The range of wavelengths was based on the technical specification of the spectrofluorometer. The fluorescence measurements were performed using quartz cuvettes with stoppers and a light path of 10 mm.

### Fluorescent measurements

In each measurement, the suspension of cells, 1 ml, was transferred into 1.5 ml eppendorf, mixed with specific chemical compound and incubated for 10 min. Then the 50 μl of the mixture of fluorescent dyes was added to the solution and 0.5 ml of that suspension was transferred to quartz cuvette to record the fluorescence signal.

### Experiments

All experiments to check the response of the cells to IRR and Non-IRR compounds were performed in cell medium DMEM with 10% FBS and 1% P/S. The total number of tested compounds was 62 (34 for IRR and 28 for Non IRR). Controls were performed in the same conditions as actual measurements but in the absence of HaCaT cells.

### Data analysis

The first data analysis problem was dimensionality reduction and feature extraction. We performed it in three steps:

1. Transformation of the set of fluorescent images into cross-correlation space;
2. Principal component analysis in the cross-correlation space;
3. Selection the principal components for retaining by usage of the Kaiser rule or the broken stick model.

At the first step we represent each fluorescence image by the vector of its correlation coefficients the images from the training set. This step reduces dimension to the number of samples in the training set. At the second step we perform the standard PCA for the samples represented by vectors of their correlation coefficients. At the third step, we select the number of principal components to retain using two well-known heuristics, the Kaiser rule (retain the principal components with the eigenvalues bigger than 1, that is the average eigenvalue of any correlation matrix) and the broken stick model. A broken stick method assumes that if the total variance (i.e., sum of the eigenvalues) is divided randomly amongst the various components, then the expected distribution of the eigenvalues will be close to a broken stick distribution. The broken stick distribution for *n* numbers is the distribution of the length of the pieces produced from an interval (a “stick”) by *n*-1 independent random cuts. Observed eigenvalues are selected as “non-random” or interpretable if they exceed eigenvalues generated by the broken-stick model^38^. The number of samples in the training set should not exceed 62 (the total number of samples available). If we use the LOOCV test then the training set always has 61 element. If we use the test set then it is less. For the test sets we select randomly 9 IRR and 10 Non-IRR compounds. For this type of testing, there remain 43 samples in the training set.

It is necessary to stress that the transformation of images into their cross-correlation space is a non-linear (bilinear) transformation on the training set because each training sample is transformed into a vector of its correlation coefficients with all the training samples. After feature extraction this transformation becomes linear on test samples. It can be represented as the set of correlation coefficients of a test image with several standardized images. We can call these standardized images “masks”. The masks for the fluorescence images with and without growing cells (for the classification task 3) are presented in Supplementary Material.

For normalized and centralized data, the transformation of the set into cross-correlation space is equivalent to the representation of each image as a vector of squared Euclidean distances to other images.

The first classifier we used was Fisher’s discriminant. It was the linear classifier with an explicit formula for the discriminating functional.

The hyperplane separates classes. There is no general explicit rule for the threshold selection beyond normality hypothesis. Therefore, was found by maximization of the sum Sensitivity + Specificity.

For improvement of classification we tested several families of nonlinear classifiers:

- *KNN.* The basic concept of KNN is: class of object is the class of a majority of its *k* nearest neighbours. We used three distances: the Euclidian distance, the Fisher’s transformed distance and adaptive distance. Moreover we used a weighted vote procedure with weighting of neighbours by one of the standard kernel function.
- *DT.* We tested methods which differ by splitting criterion, by the set of the input features, by the features used in splitting criteria, and by the minimal number of instances in leaf.
- *GM.* We tested the Gaussian mixtures which differ by the set of input features and the correction method of prior probabilities.
- *PDFE*. We tested the versions of this method which differ by the kernel function, the set of input features, and the number of nearest neighbours (between 5 and 30) used to estimate the radius of neighborhood.

- *LR* (no options).
- *NB* (the standard version, no options).
- *LDA* (described above).

The testing technology was described in detail in the supplementary material to the biomedical case study^35^.

## Additional Information

**How to cite this article**: Moczko, E. *et al.* Fluorescence-based assay as a new screening tool for toxic chemicals. *Sci. Rep.* **6**, 33922; doi: 10.1038/srep33922 (2016).

## Supplementary Material

Supplementary Materials S1

Supplementary Materials S2

## Figures and Tables

**Figure 1:**
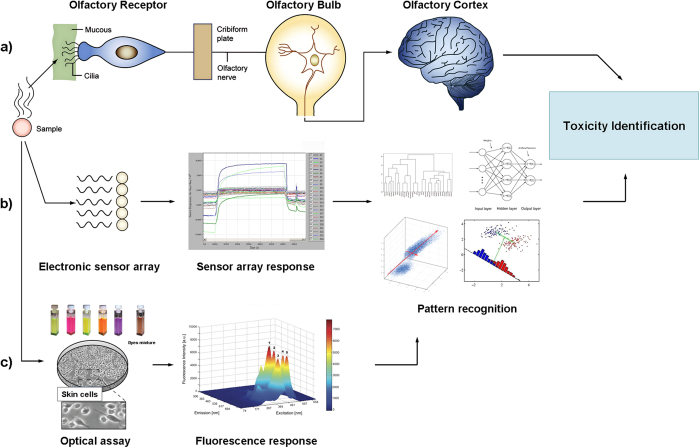
Schematic representation of sensing systems: (**a**) human olfactory system, (**b**) electronic analogue and (**c**) our optical analogue. Reprinted (adapted) with permission from (E. Moczko, I. V. Meglinski, C. Bessant and S. A. Piletsky. *Anal.Chem.*, 2009, *81* (**6**), pp 2311–2316, http://pubs.acs.org/doi/full/10.1021/ac802482h). Copyright (2009) American Chemical Society.

**Table 1:** Results of Fisher’s linear discriminant analysis for different classification tasks and all 62 compounds.

	Inputs without cells (task 1, control)	Inputs with cells (task 2)	Inputs with and without cells (task 3)
Specificity (%)	79	88	91
Sensitivity (%)	31	83	83
Sum (%)	110	171	174

**Table 2:** Classification performance for different families of methods.

Method	LOOCV (61 + 1)			Training set test (43)			Test set test (19)		
	Spec	Sens	Sum	Spec	Sens	Sum	Spec	Sens	Sum
DT	88	90	178	87	95	182	90	89	179
GM	82	76	158	91	75	166	90	78	168
3NN	88	90	181	87	90	177	90	78	168
LDA	88	83	171	91	85	176	90	89	179
LR	79	86	165	91	75	166	90	78	168
PDFE	79	86	165	96	80	176	90	78	168

Inputs with growing cells (task 2).Spec stands for Specificity (%), Sens stands for Sensitivity (%).

**Table 3:** Classification performance for different families of methods.

Method	LOOCV (61 + 1)			Training set test (43)			Test set test (19)		
	Spec	Sens	Sum	Spec	Sens	Sum	Spec	Sens	Sum
DT	91	93	184	83	95	178	70	89	159
GM	85	86	171	91	85	176	90	78	168
3NN	97	93	190	91	80	171	90	78	168
LDA	91	83	174	83	90	173	90	89	179
LR	85	86	171	87	70	157	90	78	168
PDFE	88	79	167	96	90	186	100	78	178

Inputs with and without cells (task 3). Spec stands for Specificity (%), Sens stands for Sensitivity (%).

**Table 4:** List of fluorescent dyes selected for the sensor array.

	Dye Name	Excitation [nm]	Emission [nm]	Extinction coefficient [cm^−1^M^−1^]	Supplier CAS Number
1	8–Hydroxypyrene-1′,3,6-Trisulfonic Acid	470	527	24,000 (water)	Sigma-Aldrich 6358-69-6
2	Oregon Green 514 carboxylic acid	504	528	86,000 (DMF)	Molecular Probes N/A
3	Rhodamine B	541	576	88,000 (water)	Sigma-Aldrich 81-88-9
4	Tris (4,7-diphenyl-1,10-phenanthroline) ruthenium dichloride	541	628	14600 (water)	Sigma-Aldrich 50525-27-4
5	Thionin Acetate	463	637	53,000 (water)	Sigma-Aldrich 78338-22-4
