# Supplementary material for: Fluorescence-based assay as a new screening tool for toxic chemicals
Source: Sci Rep. 2016 Sep 22;6:33922. doi: 10.1038/srep33922 (PMC5031998; doi:10.1038/srep33922)
Supplement: Supplementary Materials S1 [file srep33922-s1.pdf]

Supplementary Material

Fluorescence-based assay as a new screening tool for toxic chemicals

Ewa Moczko*, Evgeny M. Mirkes, César Cáceres, Alexander N. Gorban, Sergey Piletsky

S1. Masks of the five first principal components (PC) for the classification task 3. Inputs include both the image for compounds with growing cells ("With cells") and the control fluorescent image ("Without cells).

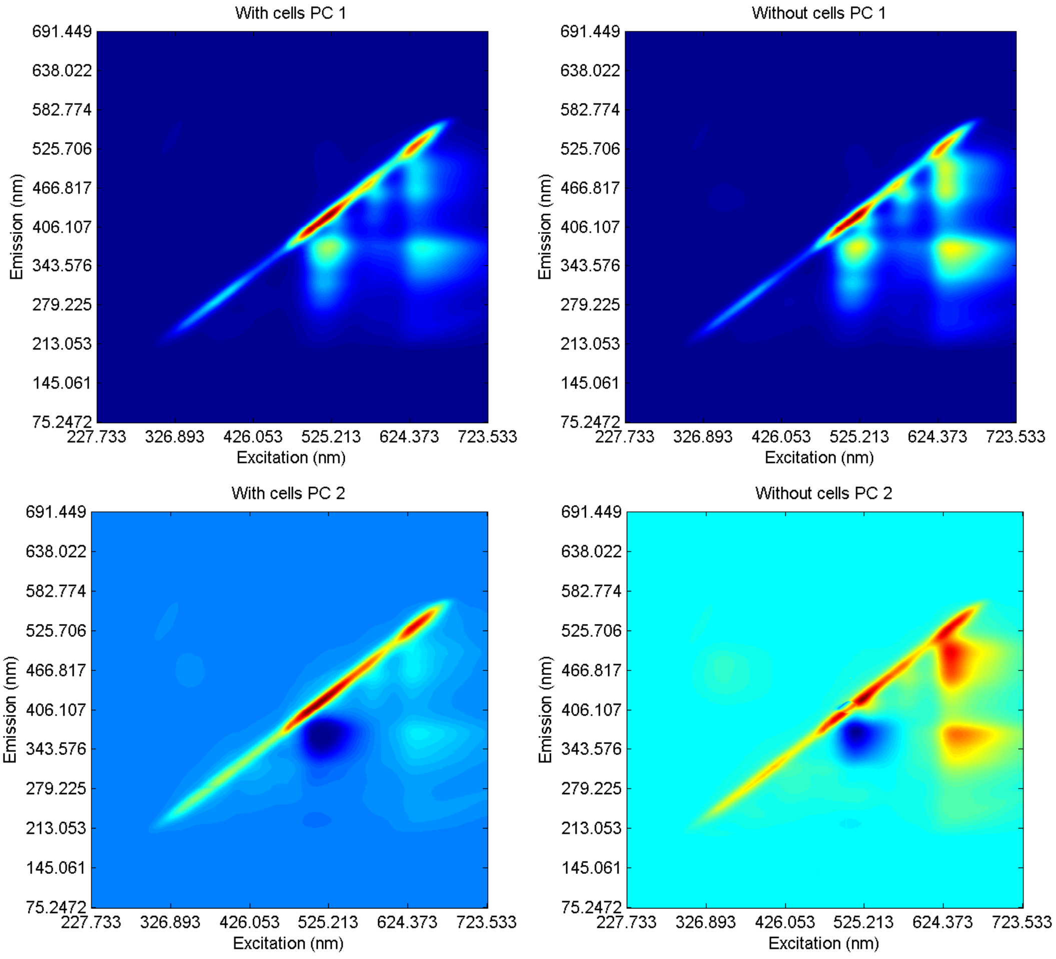

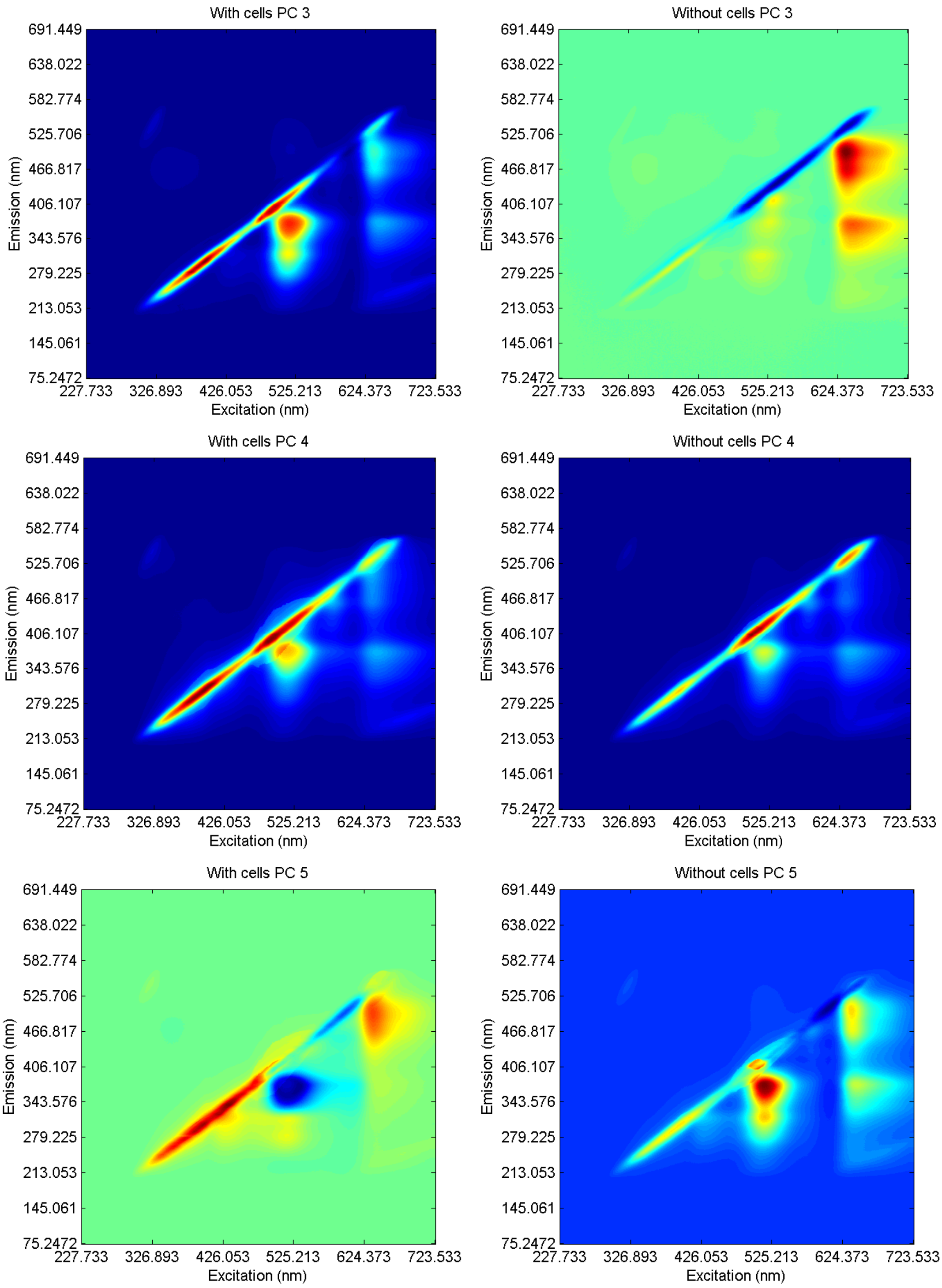
With cells PC 3
Without cells PC 3
With cells PC 4
Without cells PC 4
With cells PC 5
Without cells PC 5
Emission (nm)
691.449
638.022
582.774
525.706
466.817
406.107
343.576
279.225
213.053
145.061
75.2472
Excitation (nm)
227.733
326.893
426.053
525.213
624.373
723.533
