# Supplementary material for: Fluorescence-based assay as a new screening tool for toxic chemicals
Source: Sci Rep. 2016 Sep 22;6:33922. doi: 10.1038/srep33922 (PMC5031998; doi:10.1038/srep33922)
Supplement: Supplementary Materials S2 [file srep33922-s2.pdf]

# Supplementary Material

## Fluorescence-based assay as a new screening tool for toxic chemicals

Ewa Moczko*, Evgeny M. Mirkes, César Cáceres, Alexander N. Gorban, Sergey Piletsky

## S2. Fluorescent images. Inputs include both the image for compounds with growing cells (“With cells”) and the control fluorescent image (“Without cells).

### S2.1. Non-toxic compounds (Non-IRR)

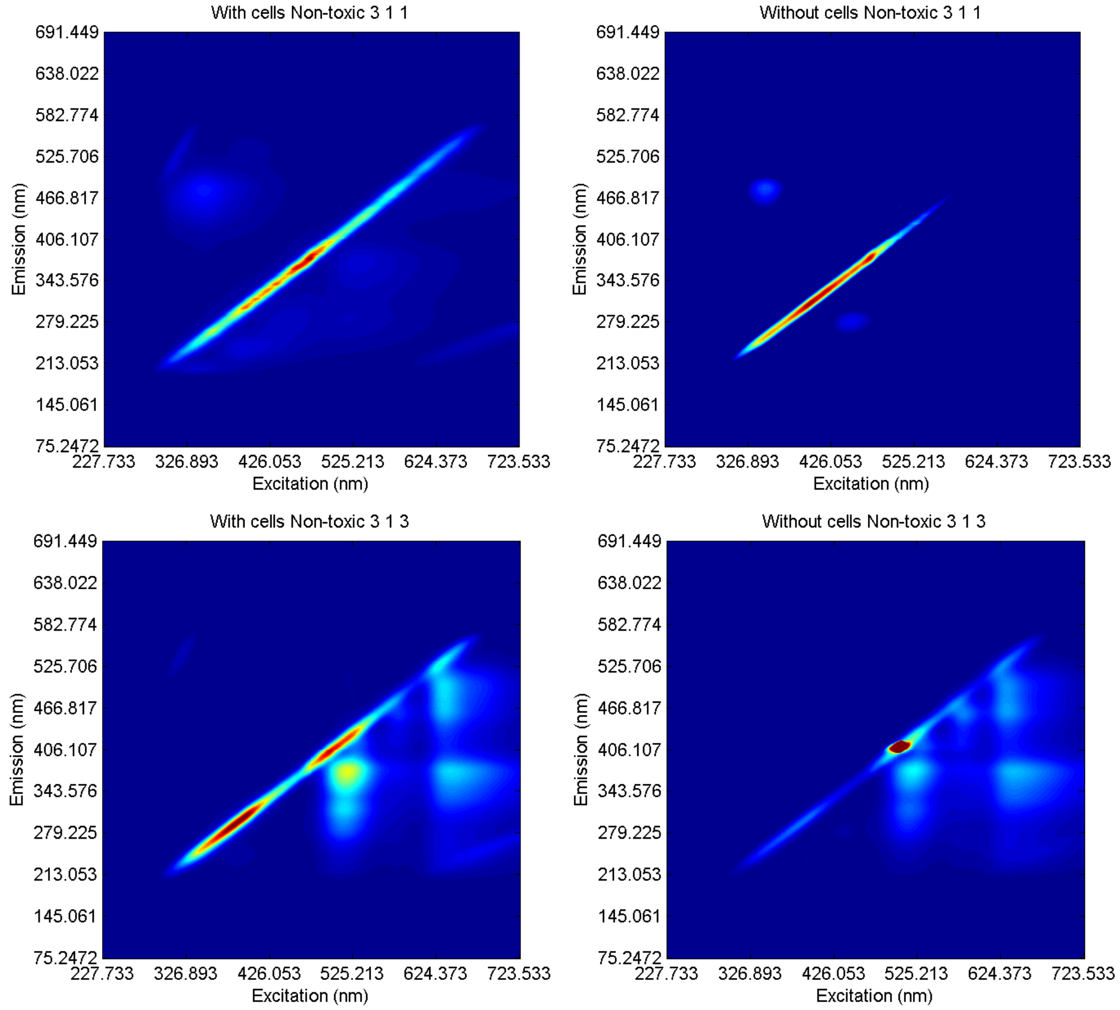

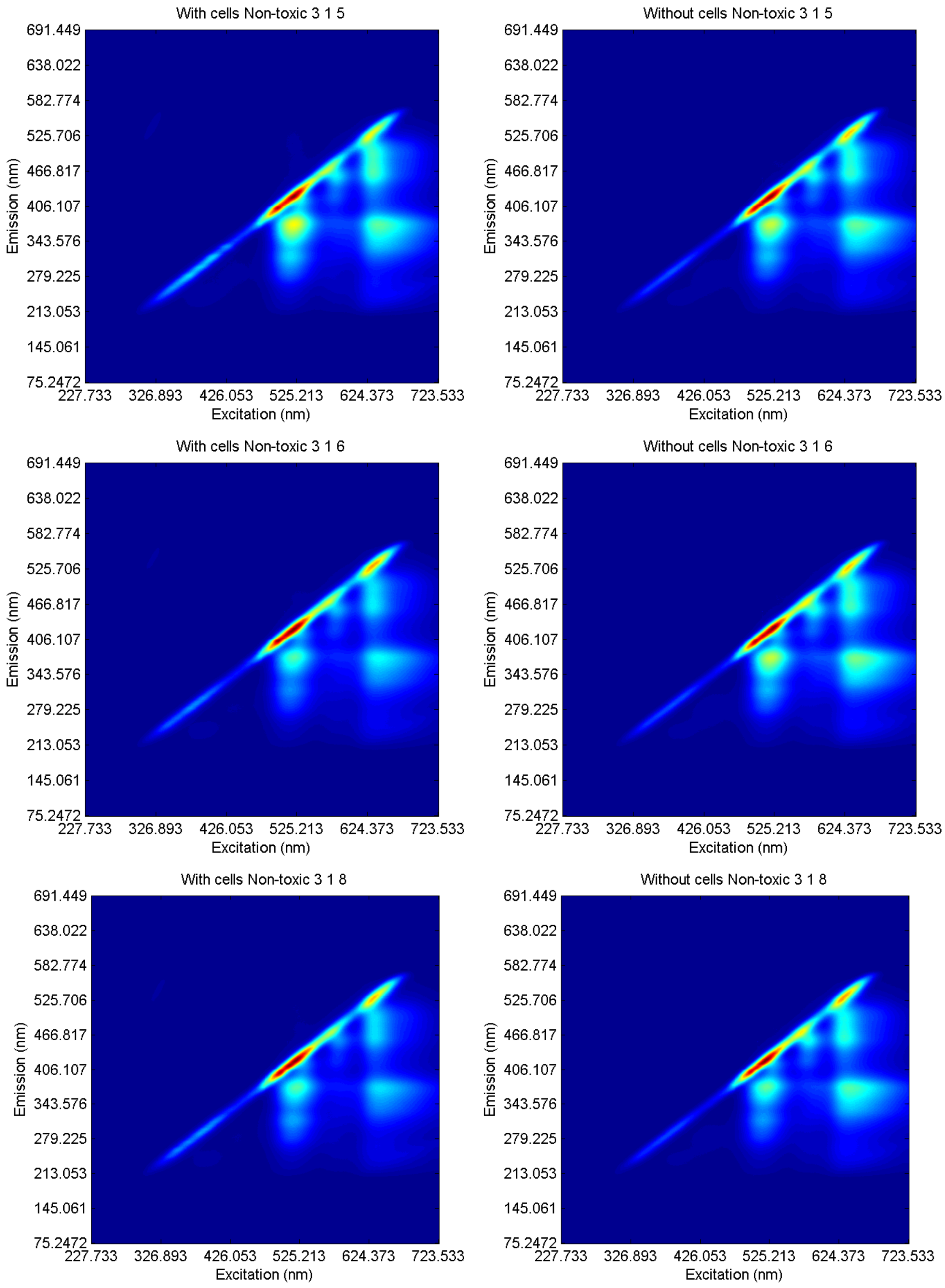
With cells Non-toxic 3 1 5
Without cells Non-toxic 3 1 5
With cells Non-toxic 3 1 6
Without cells Non-toxic 3 1 6
With cells Non-toxic 3 1 8
Without cells Non-toxic 3 1 8
Emission (nm)
691.449
638.022
582.774
525.706
466.817
406.107
343.576
279.225
213.053
145.061
75.2472
Excitation (nm)
227.733
326.893
426.053
525.213
624.373
723.533

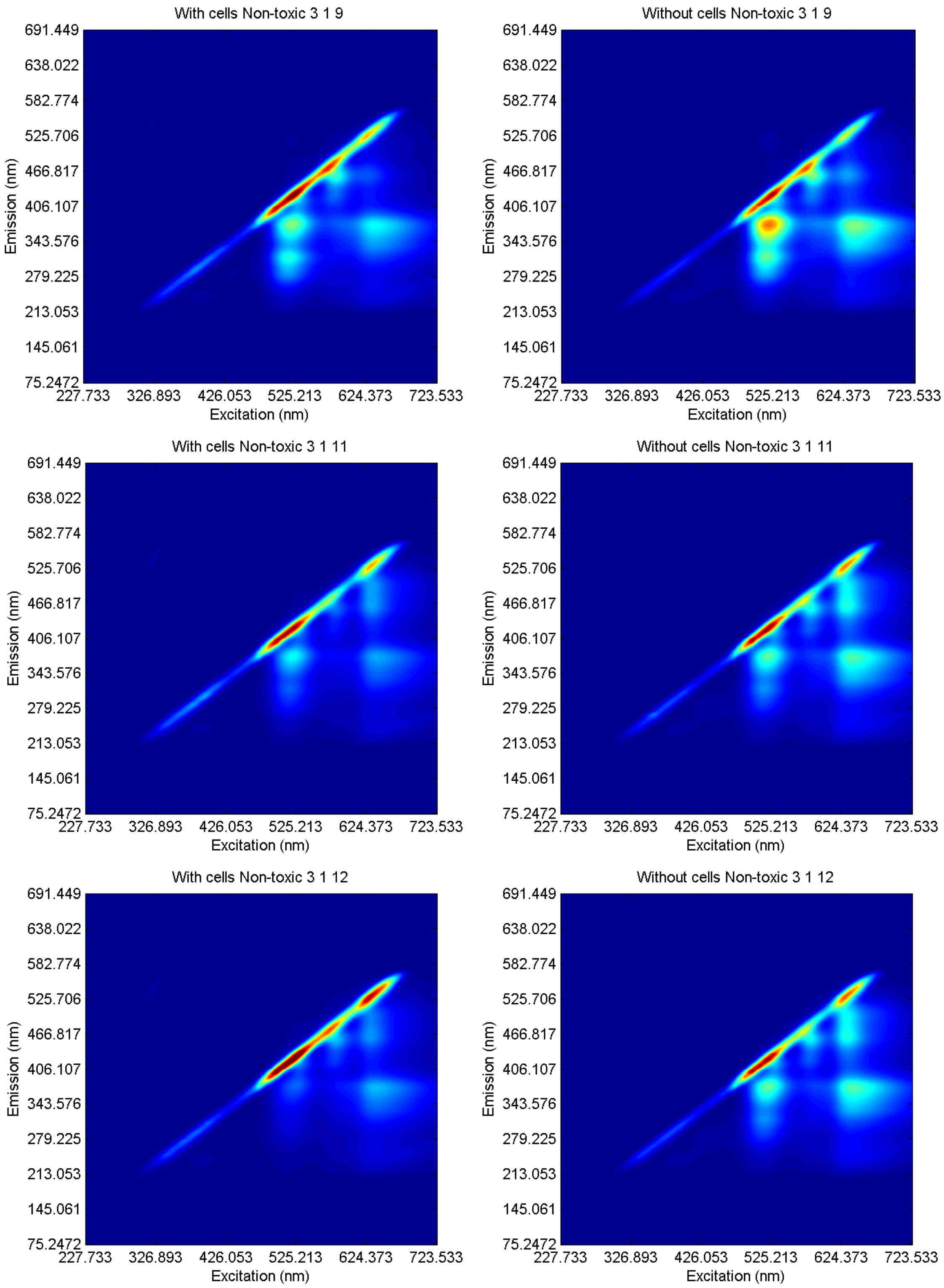
With cells Non-toxic 3 1 9
Without cells Non-toxic 3 1 9
With cells Non-toxic 3 1 11
Without cells Non-toxic 3 1 11
With cells Non-toxic 3 1 12
Without cells Non-toxic 3 1 12
Emission (nm)
691.449
638.022
582.774
525.706
466.817
406.107
343.576
279.225
213.053
145.061
75.2472
Excitation (nm)
227.733
326.893
426.053
525.213
624.373
723.533

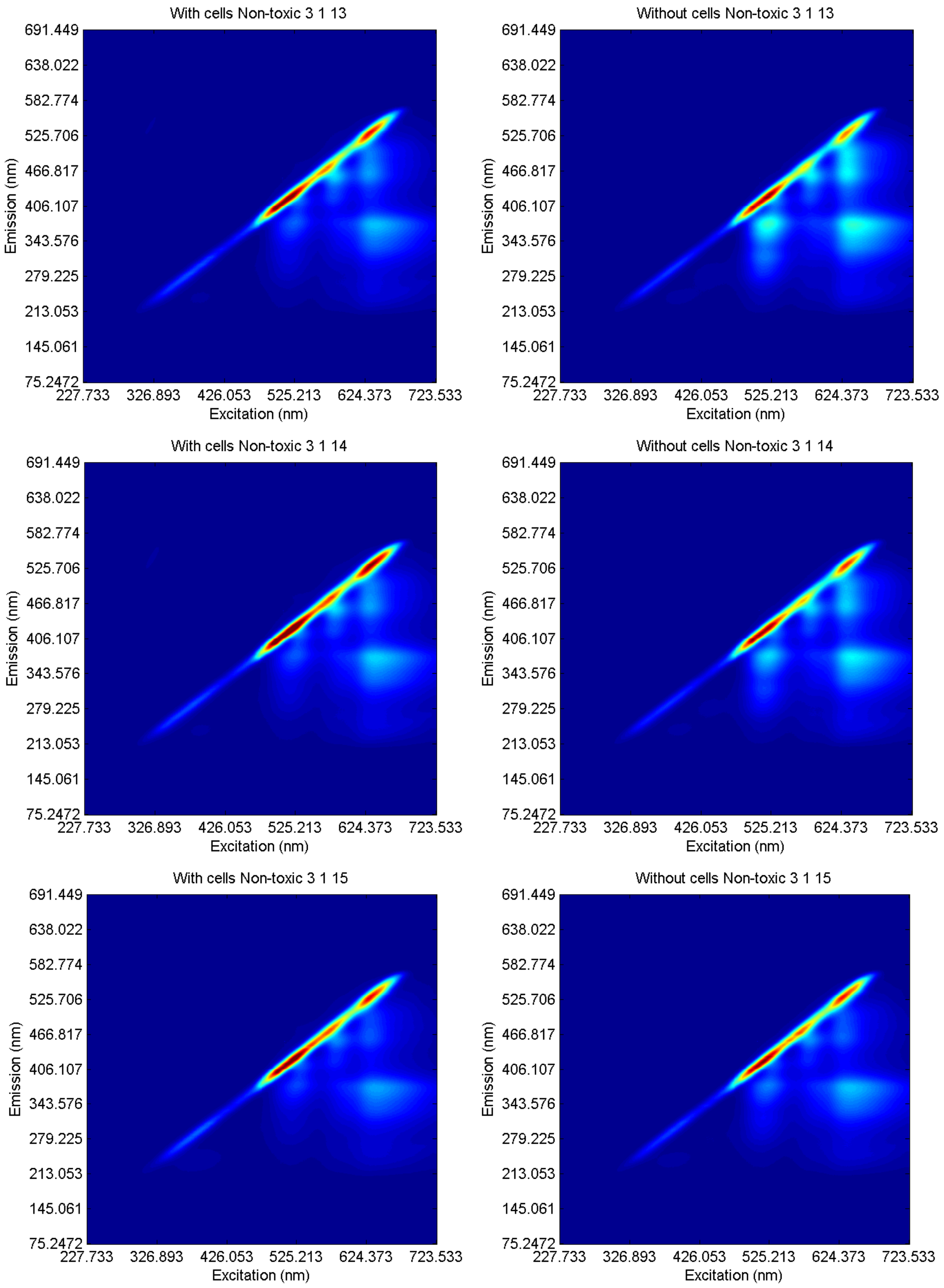
With cells Non-toxic 3 1 13
Without cells Non-toxic 3 1 13
With cells Non-toxic 3 1 14
Without cells Non-toxic 3 1 14
With cells Non-toxic 3 1 15
Without cells Non-toxic 3 1 15
Emission (nm)
691.449
638.022
582.774
525.706
466.817
406.107
343.576
279.225
213.053
145.061
75.2472
Excitation (nm)
227.733
326.893
426.053
525.213
624.373
723.533

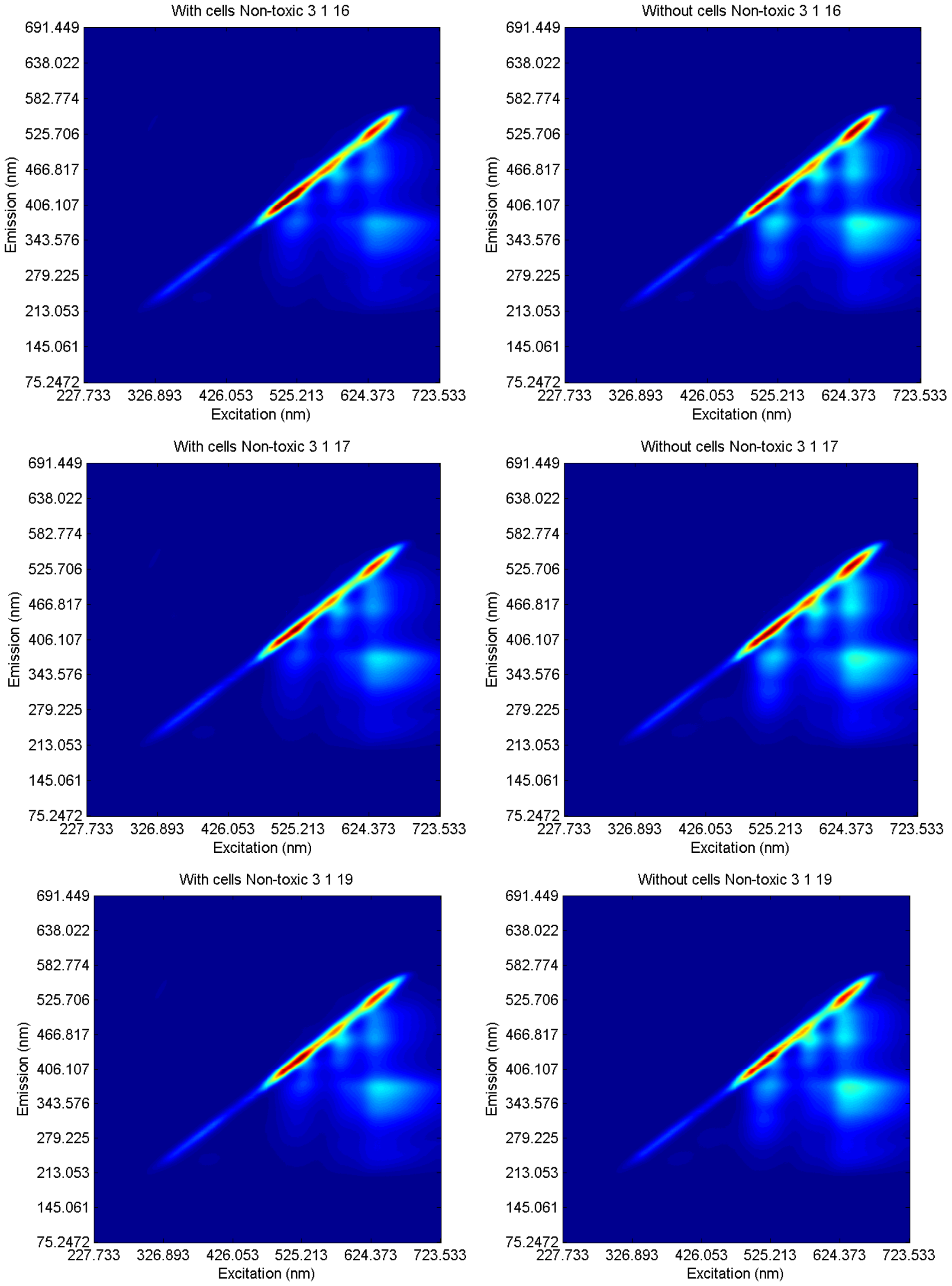
With cells Non-toxic 3 1 16
Without cells Non-toxic 3 1 16
With cells Non-toxic 3 1 17
Without cells Non-toxic 3 1 17
With cells Non-toxic 3 1 19
Without cells Non-toxic 3 1 19
Emission (nm)
Excitation (nm)
691.449
638.022
582.774
525.706
466.817
406.107
343.576
279.225
213.053
145.061
75.2472
227.733
326.893
426.053
525.213
624.373
723.533

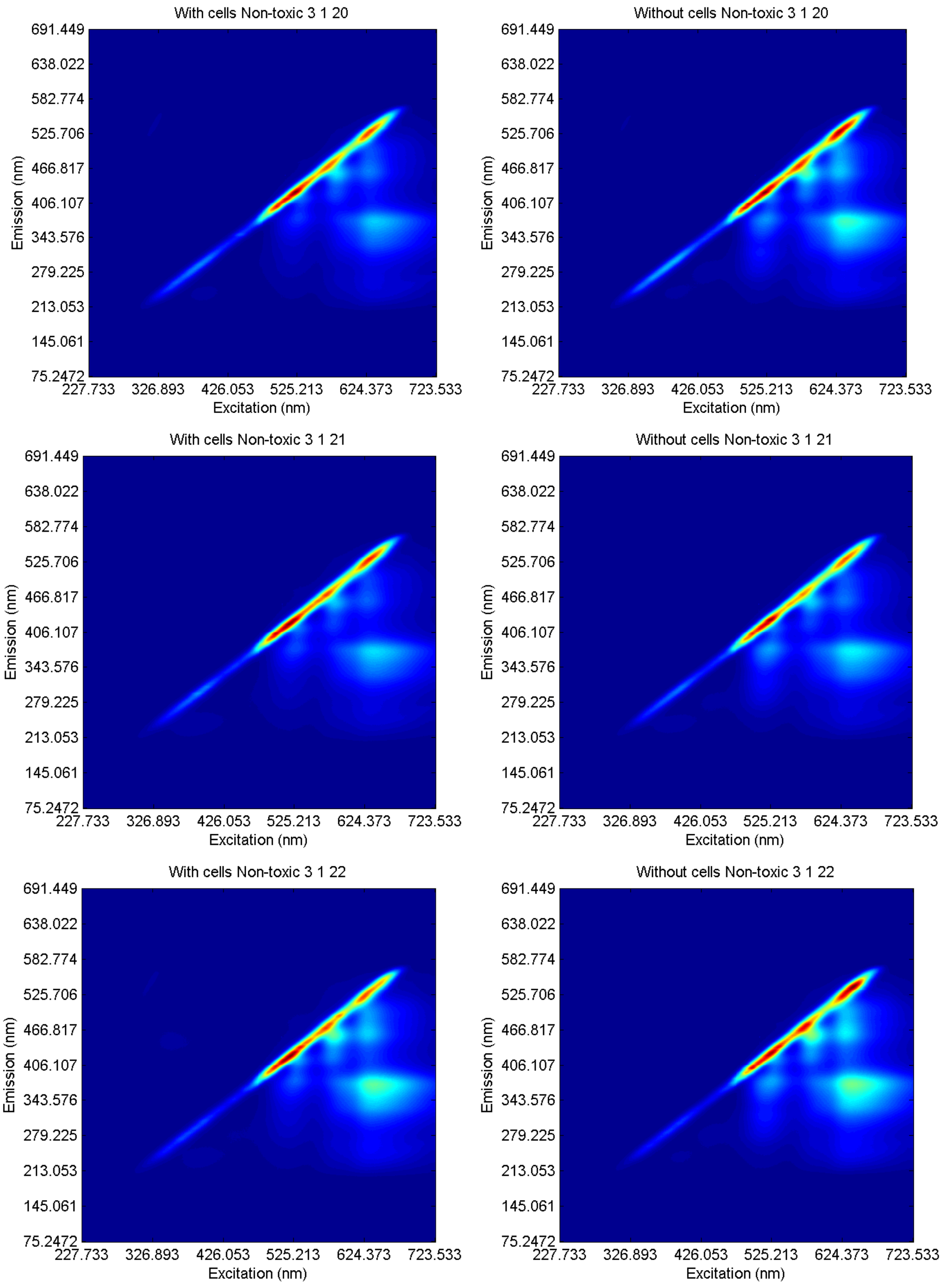

With cells Non-toxic 3 1 20
Without cells Non-toxic 3 1 20
With cells Non-toxic 3 1 21
Without cells Non-toxic 3 1 21
With cells Non-toxic 3 1 22
Without cells Non-toxic 3 1 22
Emission (nm)
691.449
638.022
582.774
525.706
466.817
406.107
343.576
279.225
213.053
145.061
75.2472
227.733
326.893
426.053
525.213
624.373
723.533
Excitation (nm)

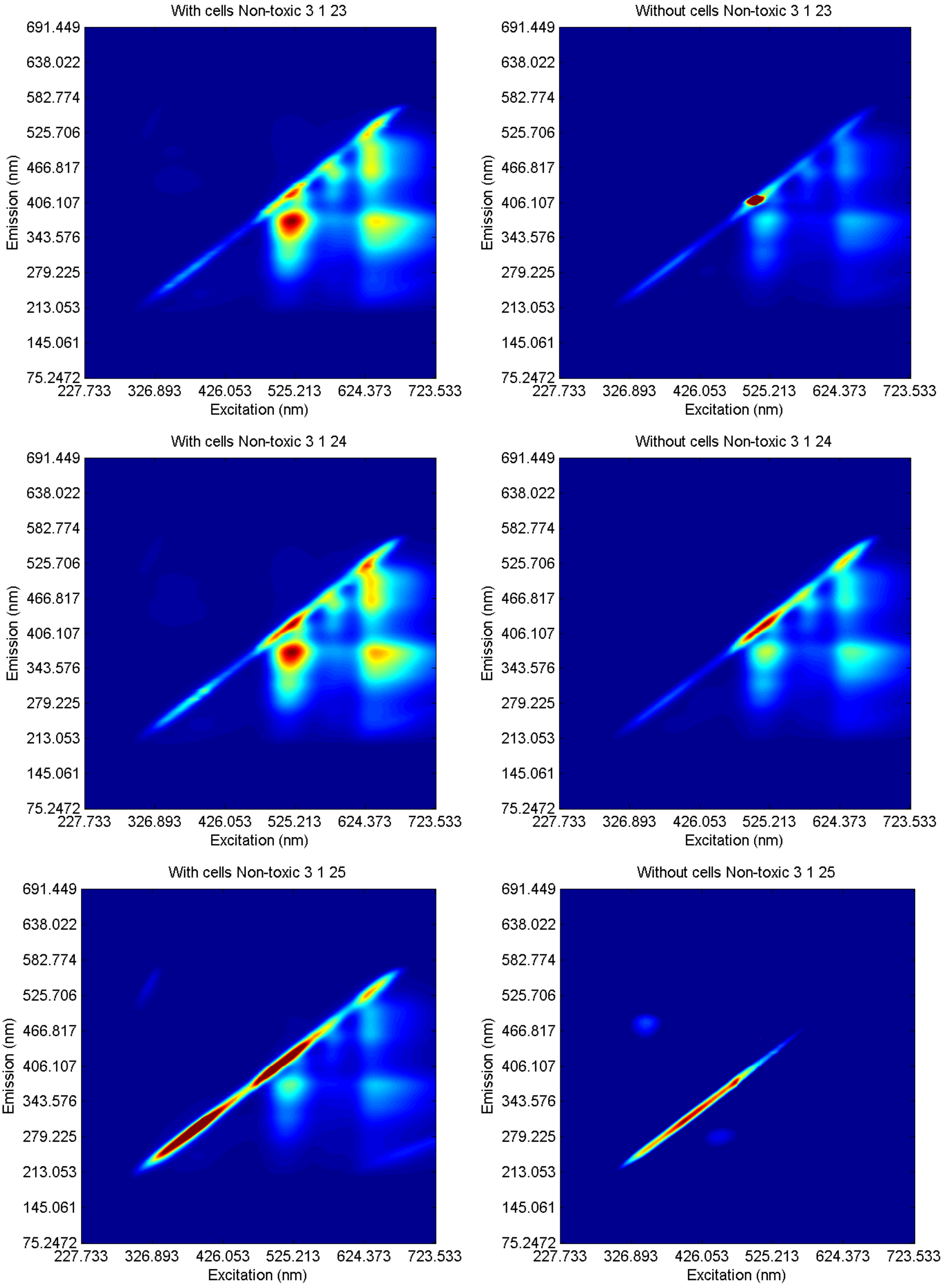
With cells Non-toxic 3 1 23
Emission (nm)
691.449
638.022
582.774
525.706
466.817
406.107
343.576
279.225
213.053
145.061
75.2472
227.733
326.893
426.053
525.213
624.373
723.533
Excitation (nm)
Without cells Non-toxic 3 1 23
Emission (nm)
691.449
638.022
582.774
525.706
466.817
406.107
343.576
279.225
213.053
145.061
75.2472
227.733
326.893
426.053
525.213
624.373
723.533
Excitation (nm)
With cells Non-toxic 3 1 24
Emission (nm)
691.449
638.022
582.774
525.706
466.817
406.107
343.576
279.225
213.053
145.061
75.2472
227.733
326.893
426.053
525.213
624.373
723.533
Excitation (nm)
Without cells Non-toxic 3 1 24
Emission (nm)
691.449
638.022
582.774
525.706
466.817
406.107
343.576
279.225
213.053
145.061
75.2472
227.733
326.893
426.053
525.213
624.373
723.533
Excitation (nm)
With cells Non-toxic 3 1 25
Emission (nm)
691.449
638.022
582.774
525.706
466.817
406.107
343.576
279.225
213.053
145.061
75.2472
227.733
326.893
426.053
525.213
624.373
723.533
Excitation (nm)
Without cells Non-toxic 3 1 25
Emission (nm)
691.449
638.022
582.774
525.706
466.817
406.107
343.576
279.225
213.053
145.061
75.2472
227.733
326.893
426.053
525.213
624.373
723.533
Excitation (nm)

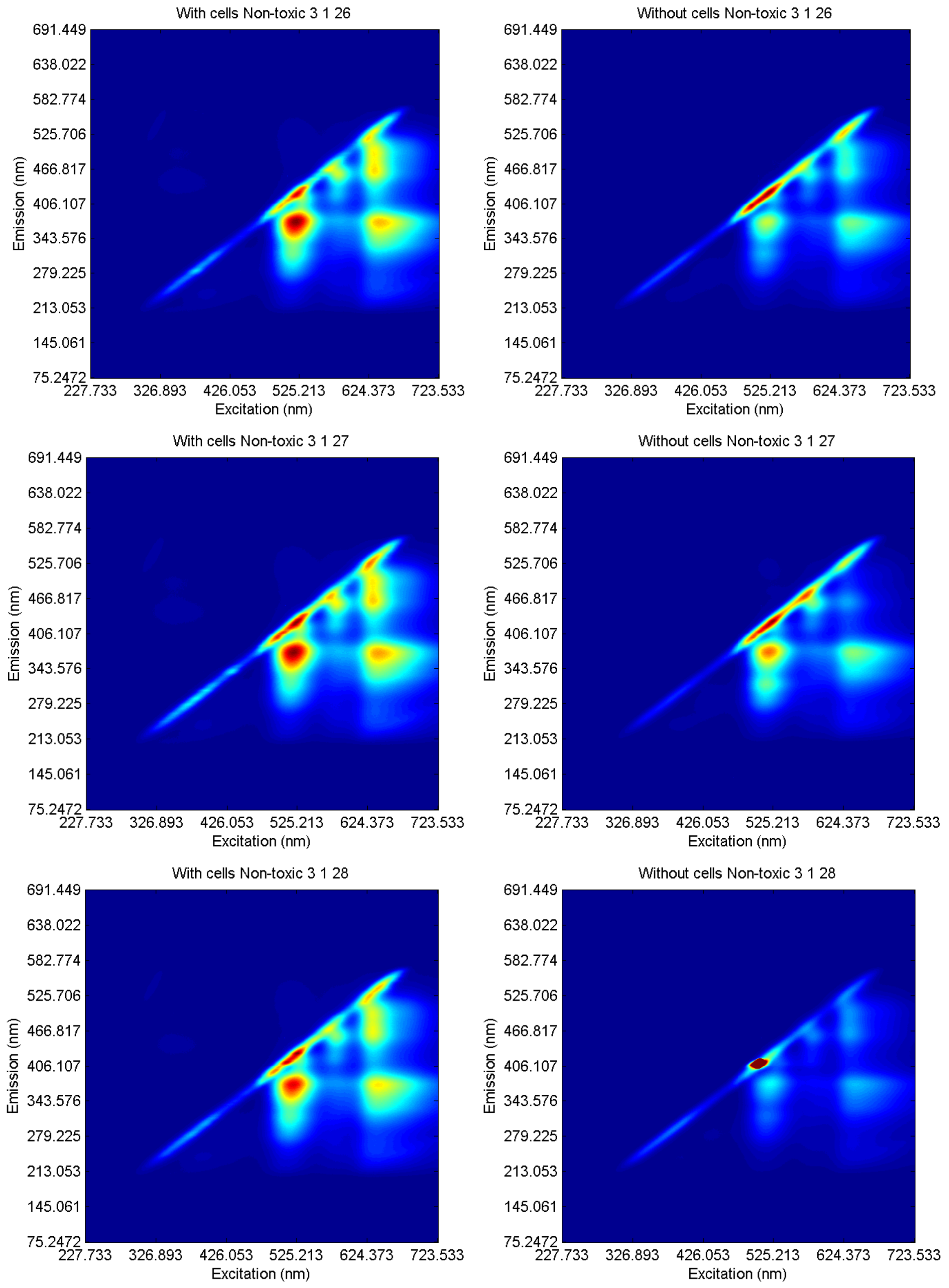
With cells Non-toxic 3 1 26
Without cells Non-toxic 3 1 26
With cells Non-toxic 3 1 27
Without cells Non-toxic 3 1 27
With cells Non-toxic 3 1 28
Without cells Non-toxic 3 1 28
Emission (nm)
691.449
638.022
582.774
525.706
466.817
406.107
343.576
279.225
213.053
145.061
75.2472
227.733
326.893
426.053
525.213
624.373
723.533
Excitation (nm)

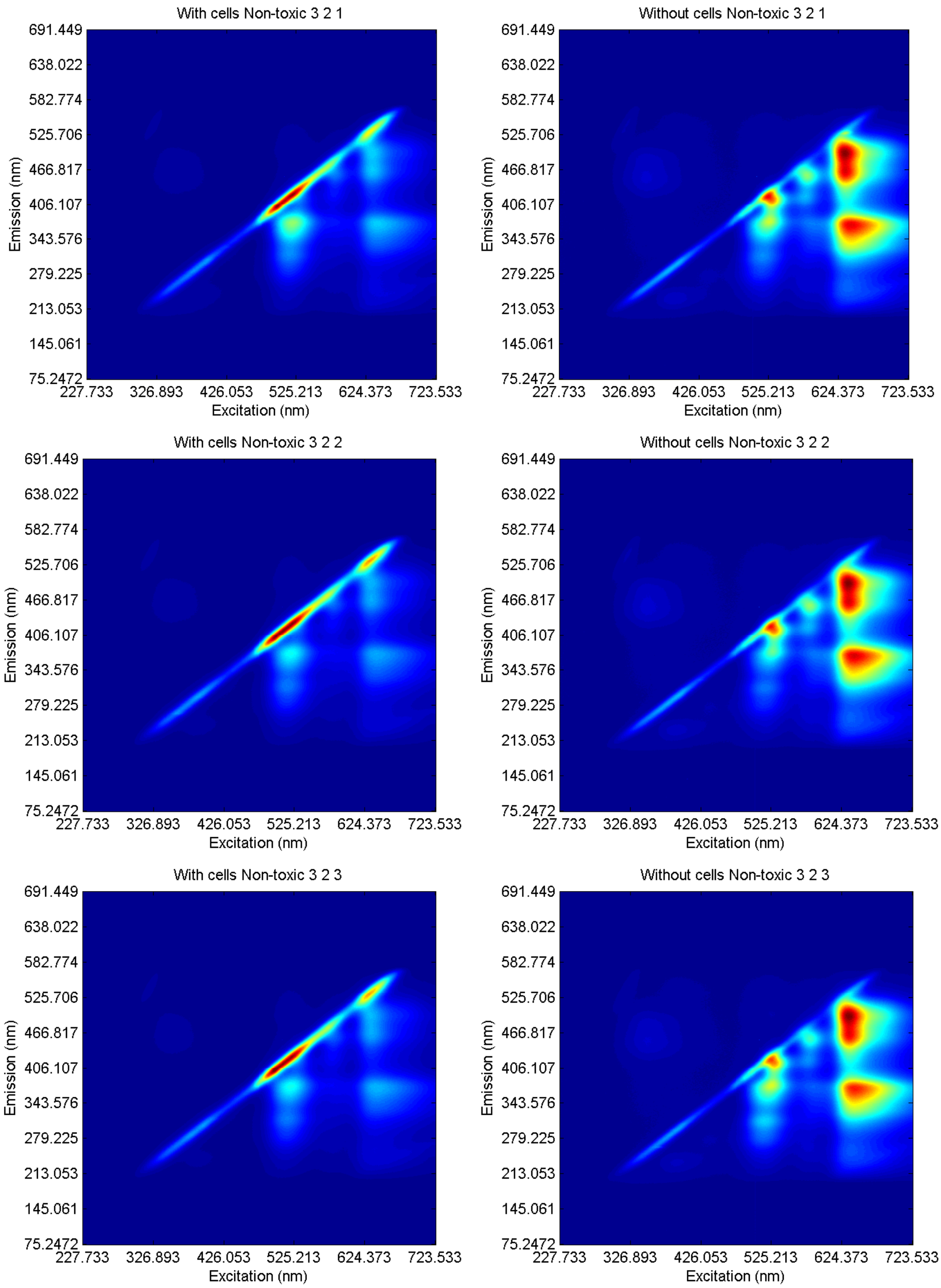
With cells Non-toxic 3 2 1
Without cells Non-toxic 3 2 1
With cells Non-toxic 3 2 2
Without cells Non-toxic 3 2 2
With cells Non-toxic 3 2 3
Without cells Non-toxic 3 2 3
Emission (nm)
691.449
638.022
582.774
525.706
466.817
406.107
343.576
279.225
213.053
145.061
75.2472
227.733
326.893
426.053
525.213
624.373
723.533
Excitation (nm)

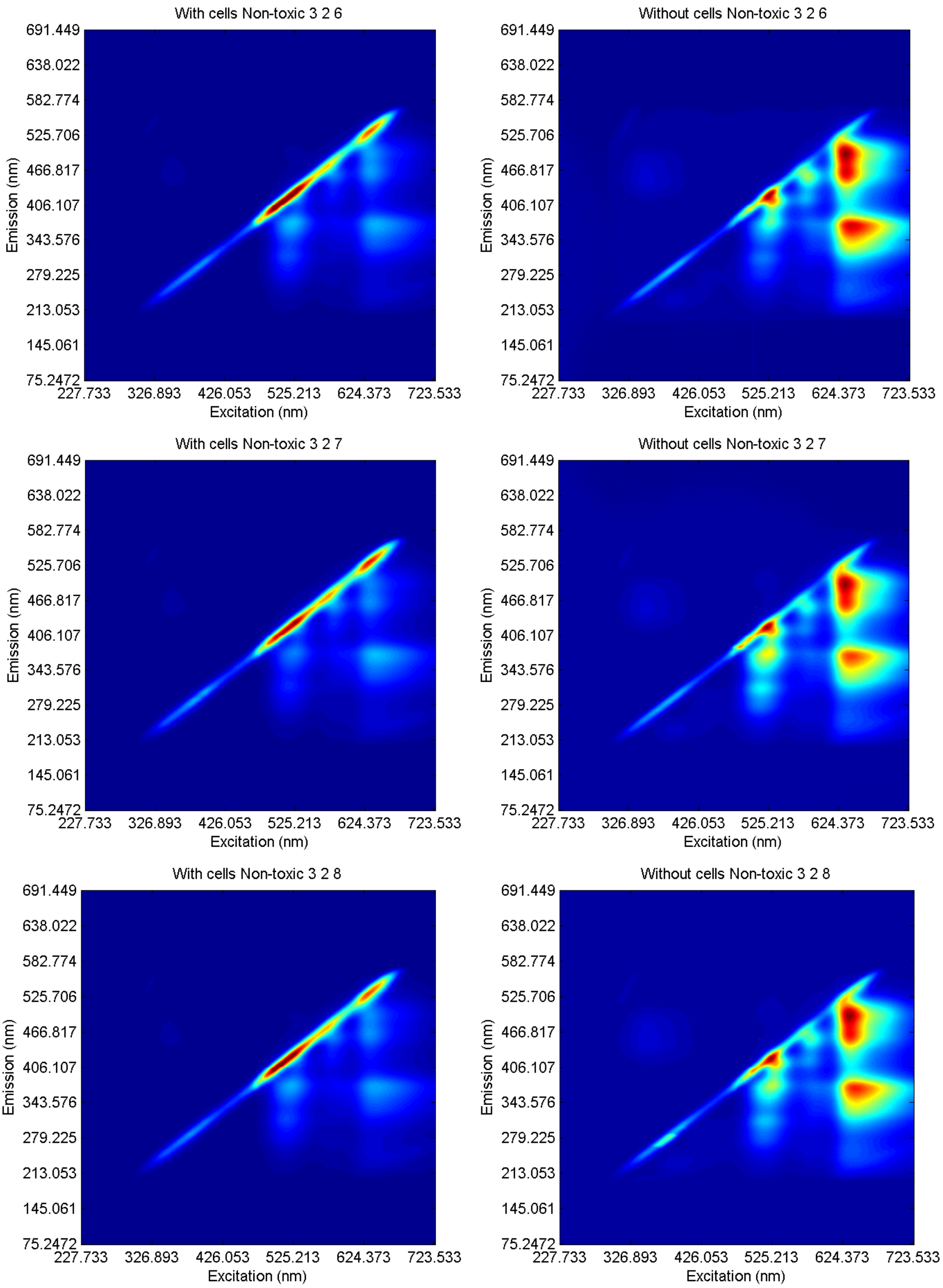

With cells Non-toxic 3 2 6
Without cells Non-toxic 3 2 6
With cells Non-toxic 3 2 7
Without cells Non-toxic 3 2 7
With cells Non-toxic 3 2 8
Without cells Non-toxic 3 2 8
Emission (nm)
691.449
638.022
582.774
525.706
466.817
406.107
343.576
279.225
213.053
145.061
75.2472
Excitation (nm)
227.733
326.893
426.053
525.213
624.373
723.533

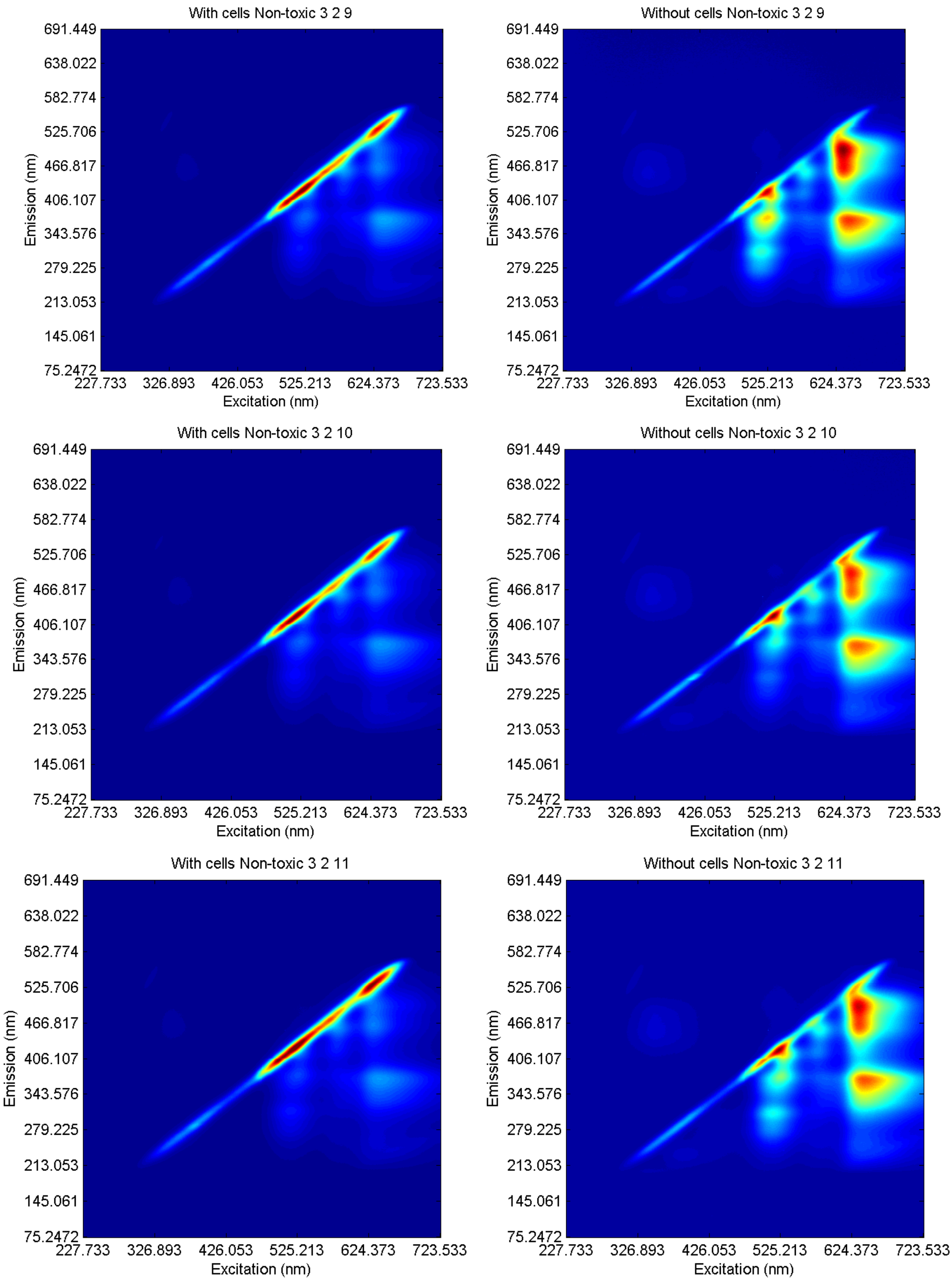
With cells Non-toxic 3 2 9
Without cells Non-toxic 3 2 9
With cells Non-toxic 3 2 10
Without cells Non-toxic 3 2 10
With cells Non-toxic 3 2 11
Without cells Non-toxic 3 2 11
Emission (nm)
691.449
638.022
582.774
525.706
466.817
406.107
343.576
279.225
213.053
145.061
75.2472
Excitation (nm)
227.733
326.893
426.053
525.213
624.373
723.533

## S2.2. Toxic compounds (IRR)

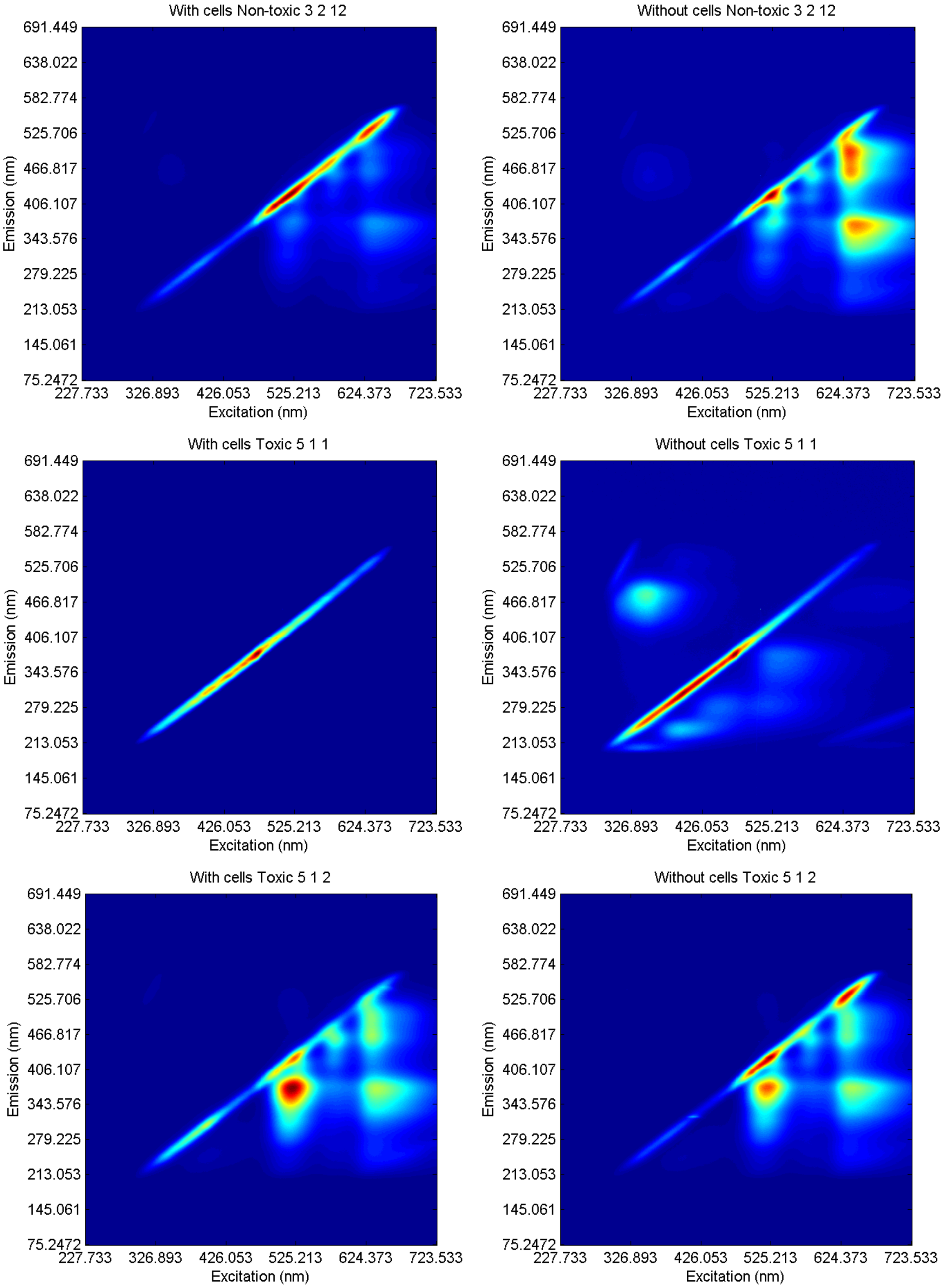

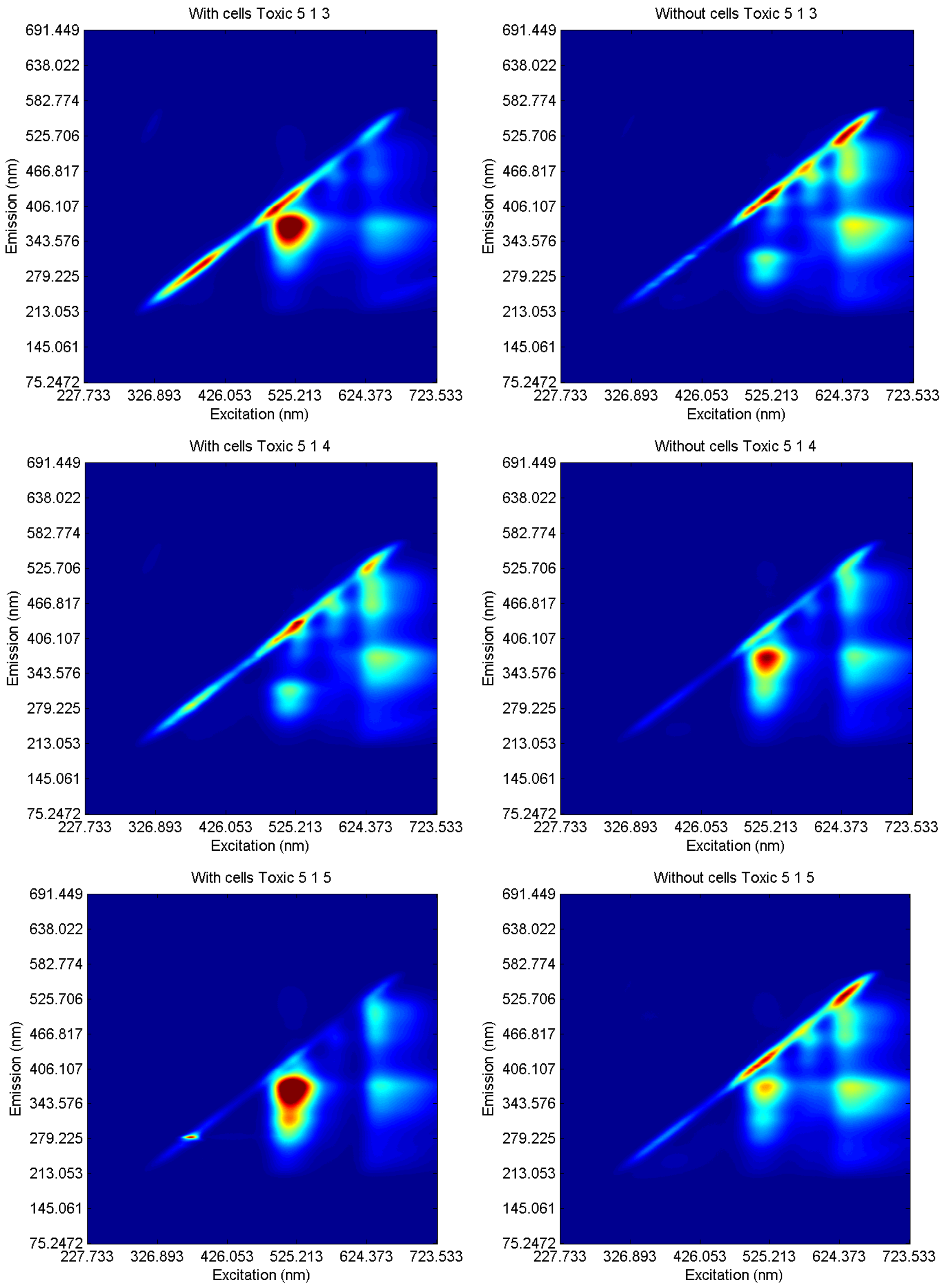
With cells Toxic 5 1 3
Without cells Toxic 5 1 3
With cells Toxic 5 1 4
Without cells Toxic 5 1 4
With cells Toxic 5 1 5
Without cells Toxic 5 1 5
Emission (nm)
691.449
638.022
582.774
525.706
466.817
406.107
343.576
279.225
213.053
145.061
75.2472
227.733
326.893
426.053
525.213
624.373
723.533
Excitation (nm)

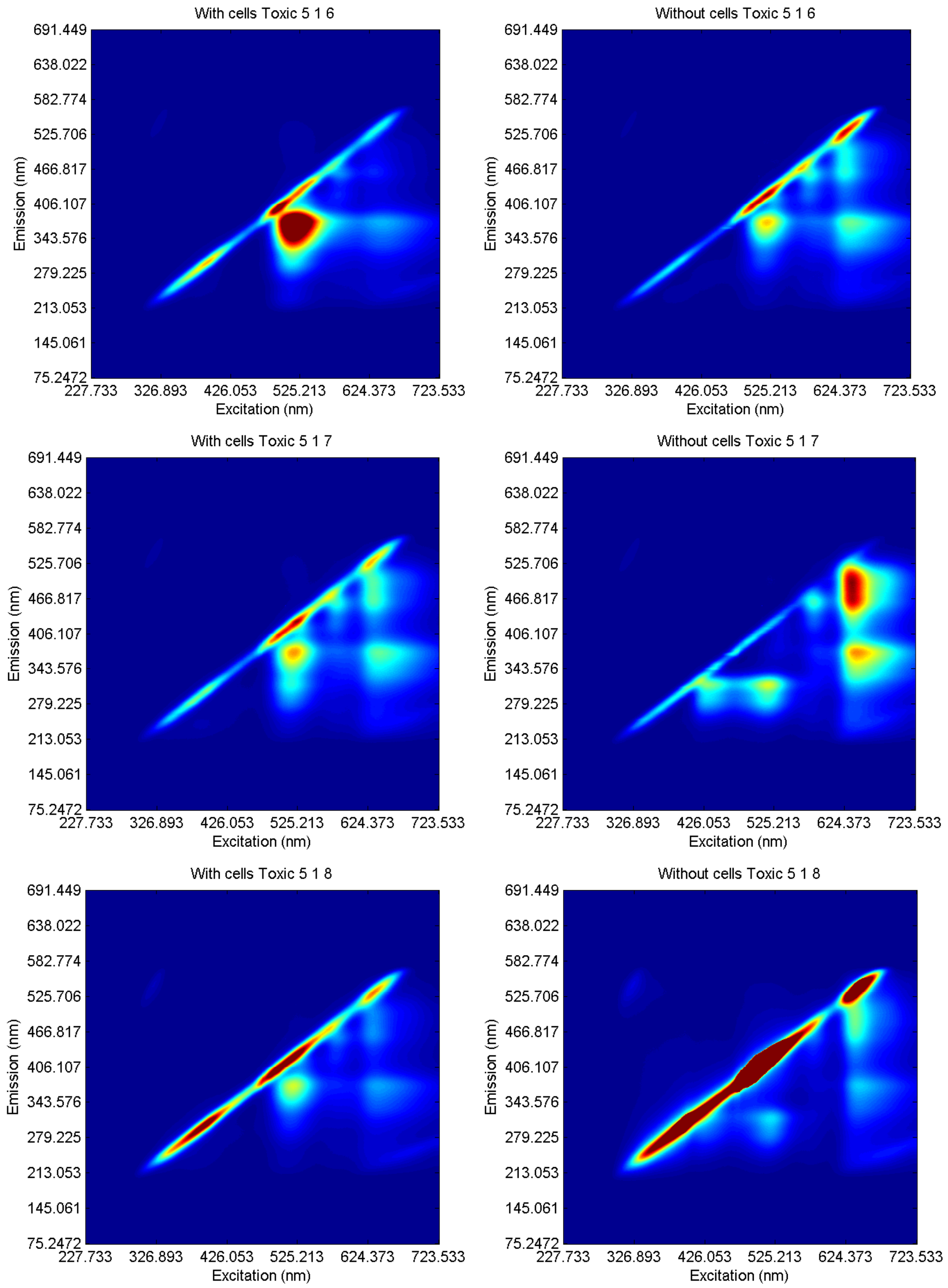

With cells Toxic 5 1 6
Without cells Toxic 5 1 6
With cells Toxic 5 1 7
Without cells Toxic 5 1 7
With cells Toxic 5 1 8
Without cells Toxic 5 1 8
691.449
638.022
582.774
525.706
466.817
406.107
343.576
279.225
213.053
145.061
75.2472
Emission (nm)
227.733
326.893
426.053
525.213
624.373
723.533
Excitation (nm)

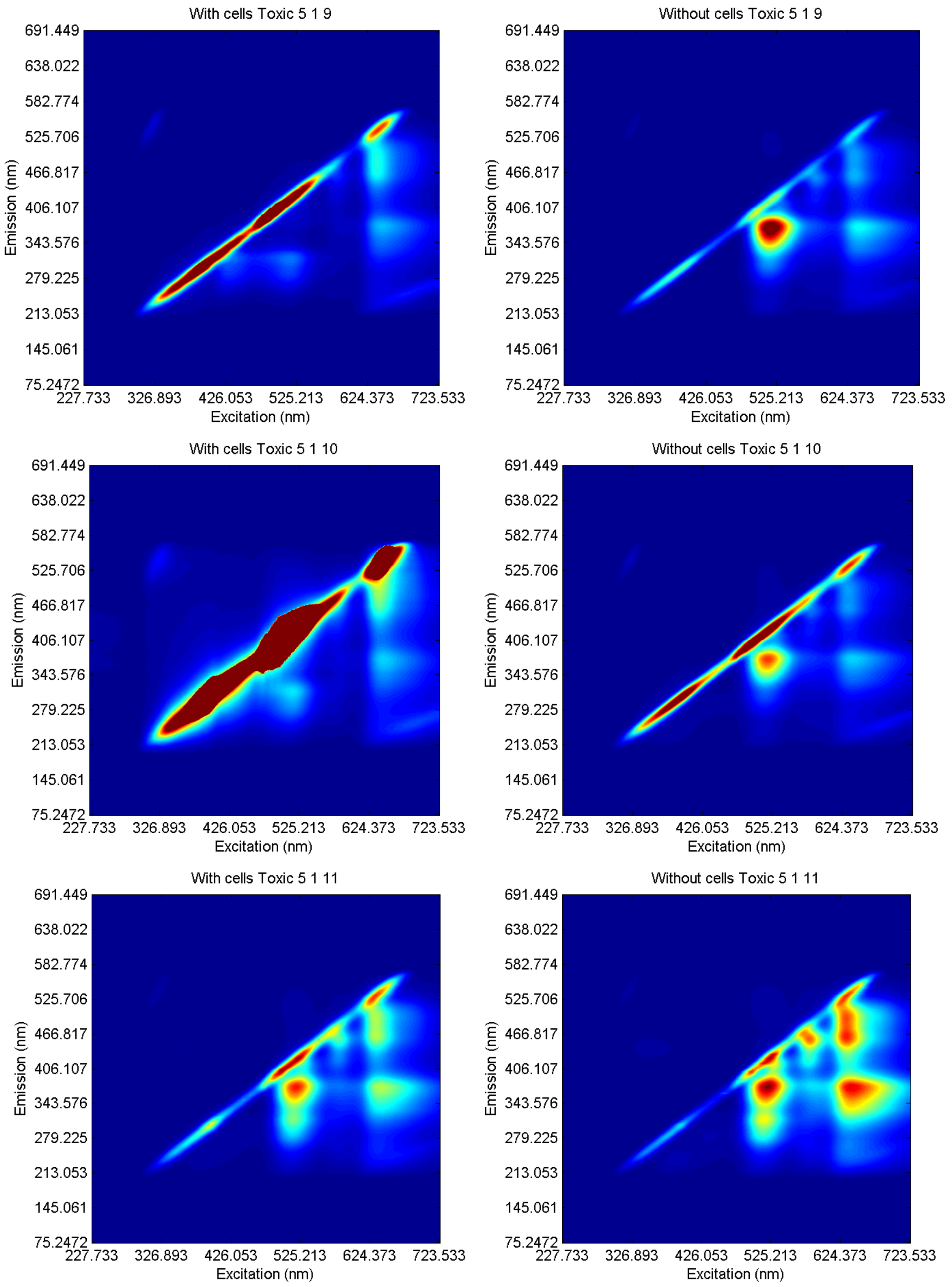
With cells Toxic 5 1 9
Without cells Toxic 5 1 9
With cells Toxic 5 1 10
Without cells Toxic 5 1 10
With cells Toxic 5 1 11
Without cells Toxic 5 1 11
Emission (nm)
Excitation (nm)
691.449
638.022
582.774
525.706
466.817
406.107
343.576
279.225
213.053
145.061
75.2472
227.733
326.893
426.053
525.213
624.373
723.533

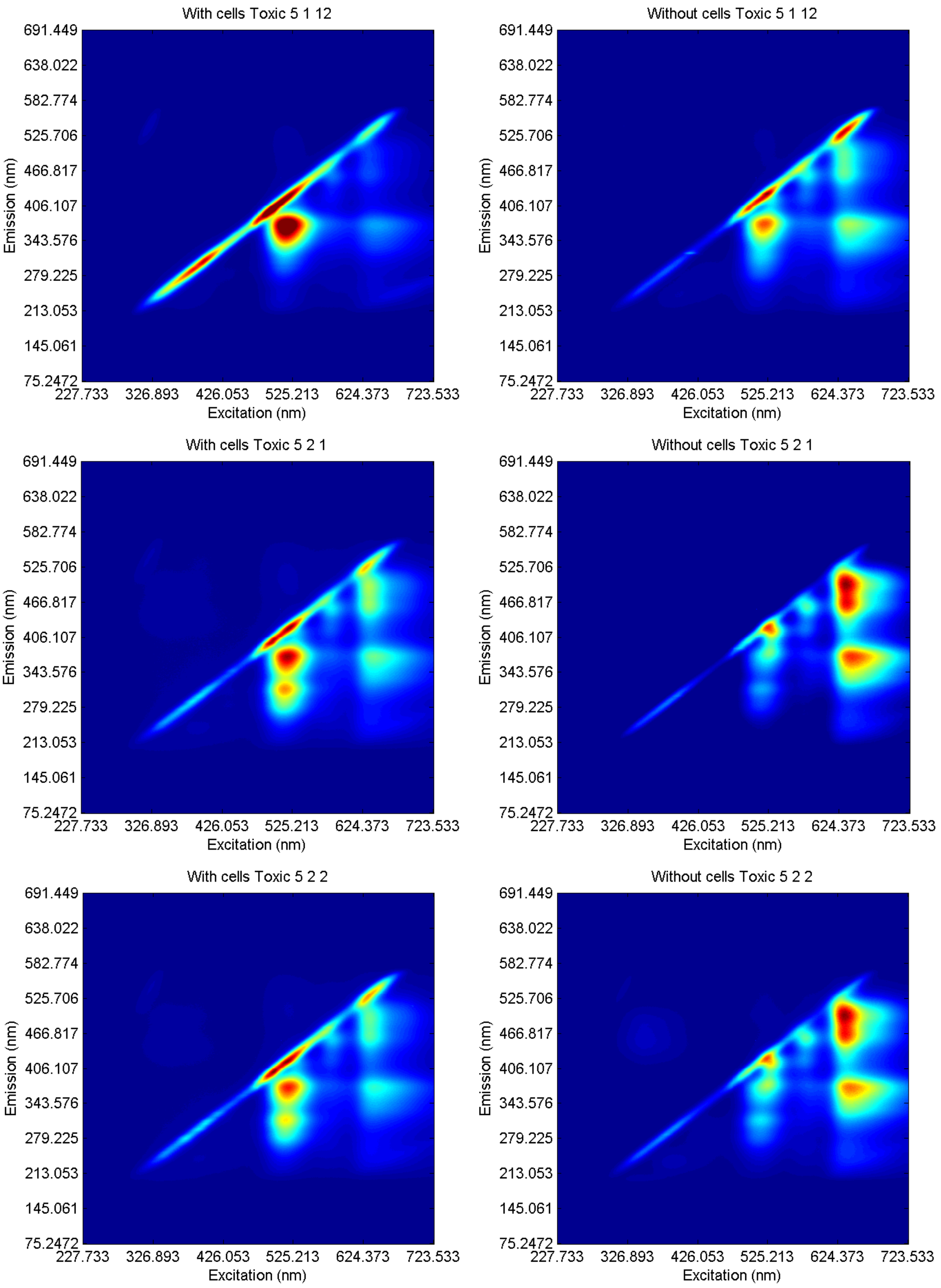
With cells Toxic 5 1 12
Without cells Toxic 5 1 12
With cells Toxic 5 2 1
Without cells Toxic 5 2 1
With cells Toxic 5 2 2
Without cells Toxic 5 2 2
Emission (nm)
691.449
638.022
582.774
525.706
466.817
406.107
343.576
279.225
213.053
145.061
75.2472
Excitation (nm)
227.733
326.893
426.053
525.213
624.373
723.533

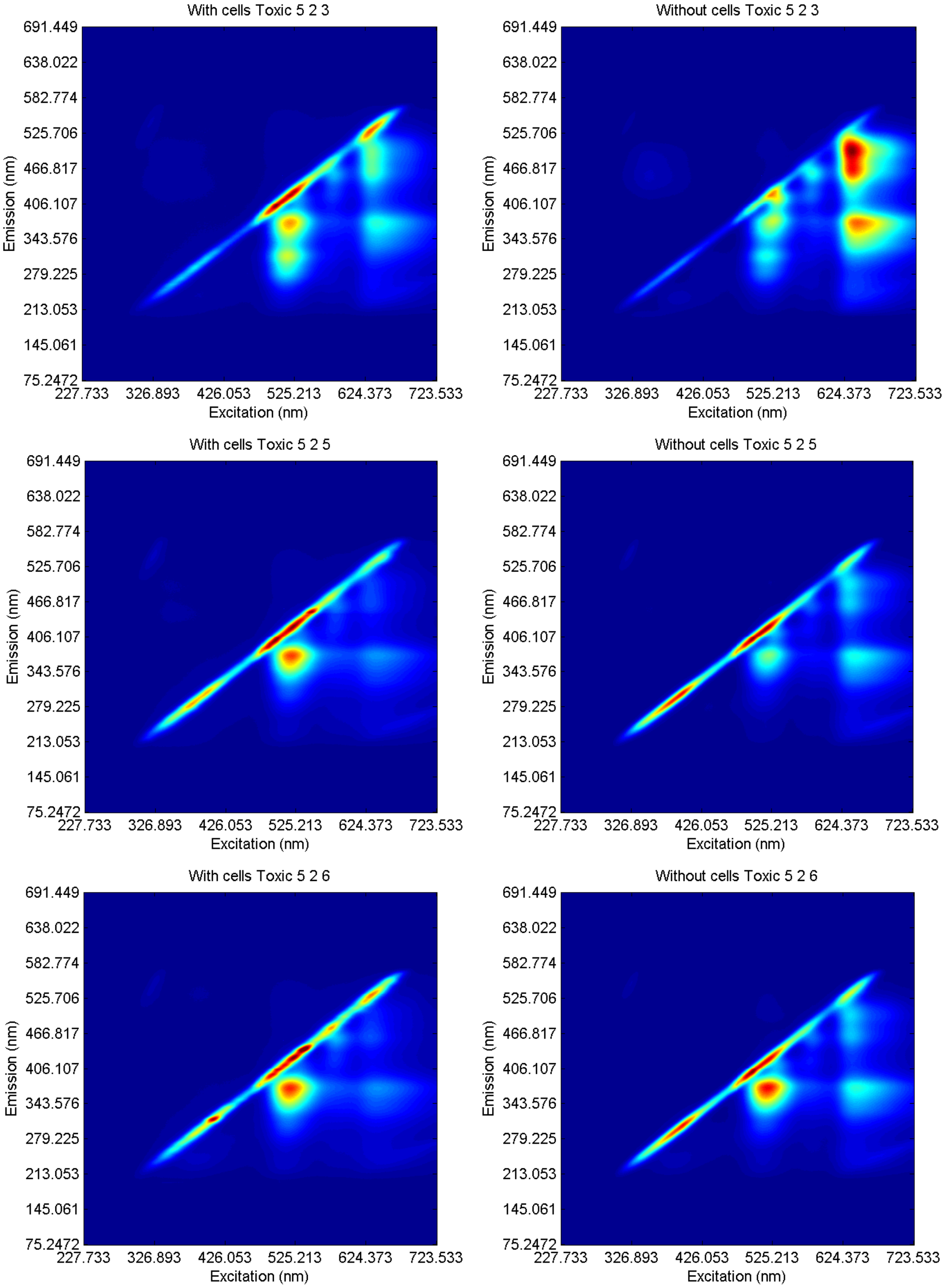

With cells Toxic 5 2 3
Without cells Toxic 5 2 3
With cells Toxic 5 2 5
Without cells Toxic 5 2 5
With cells Toxic 5 2 6
Without cells Toxic 5 2 6
Emission (nm)
691.449
638.022
582.774
525.706
466.817
406.107
343.576
279.225
213.053
145.061
75.2472
Excitation (nm)
227.733
326.893
426.053
525.213
624.373
723.533

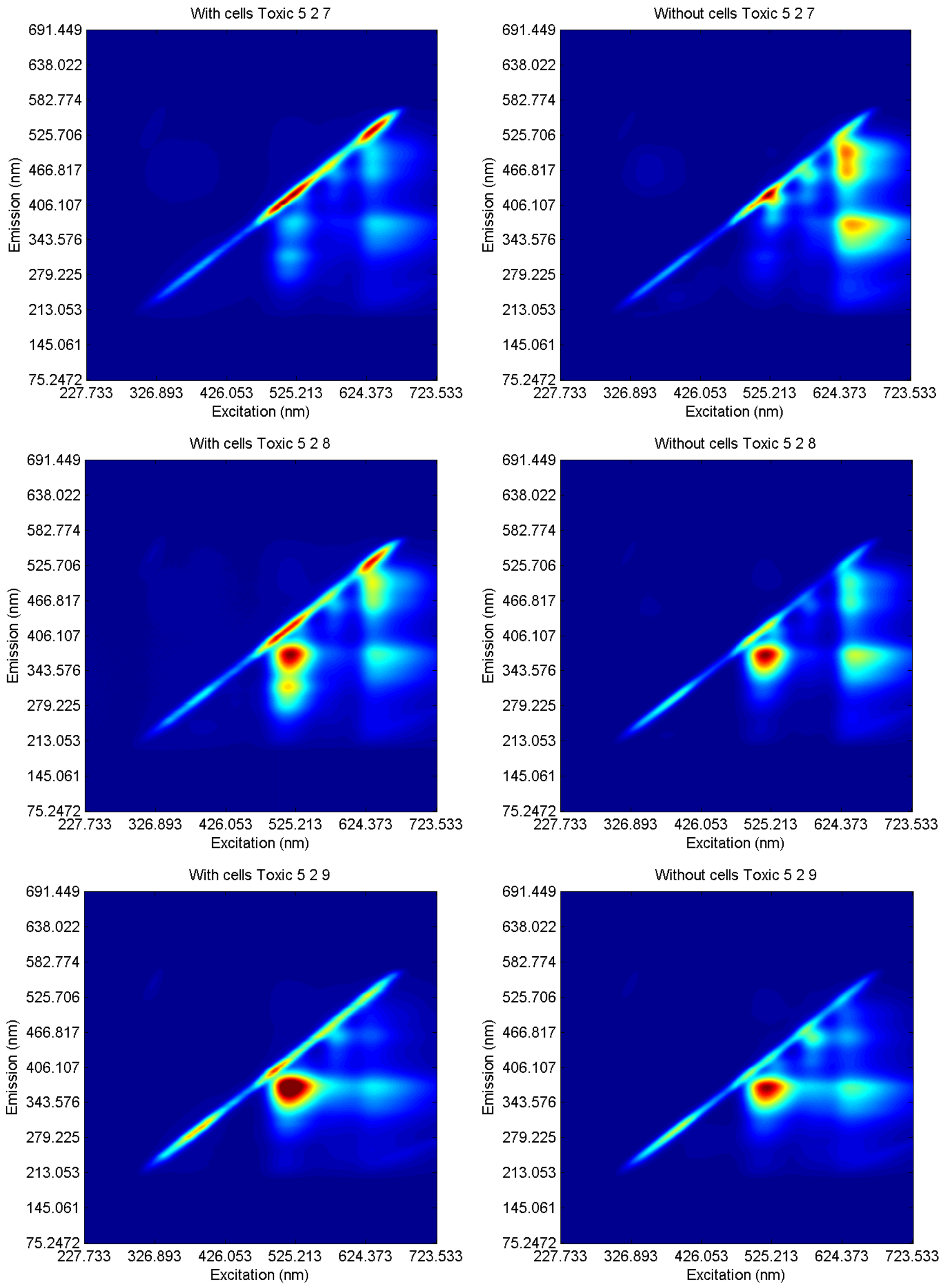

With cells Toxic 5 2 7
Without cells Toxic 5 2 7
With cells Toxic 5 2 8
Without cells Toxic 5 2 8
With cells Toxic 5 2 9
Without cells Toxic 5 2 9
Emission (nm)
691.449
638.022
582.774
525.706
466.817
406.107
343.576
279.225
213.053
145.061
75.2472
227.733
326.893
426.053
525.213
624.373
723.533
Excitation (nm)

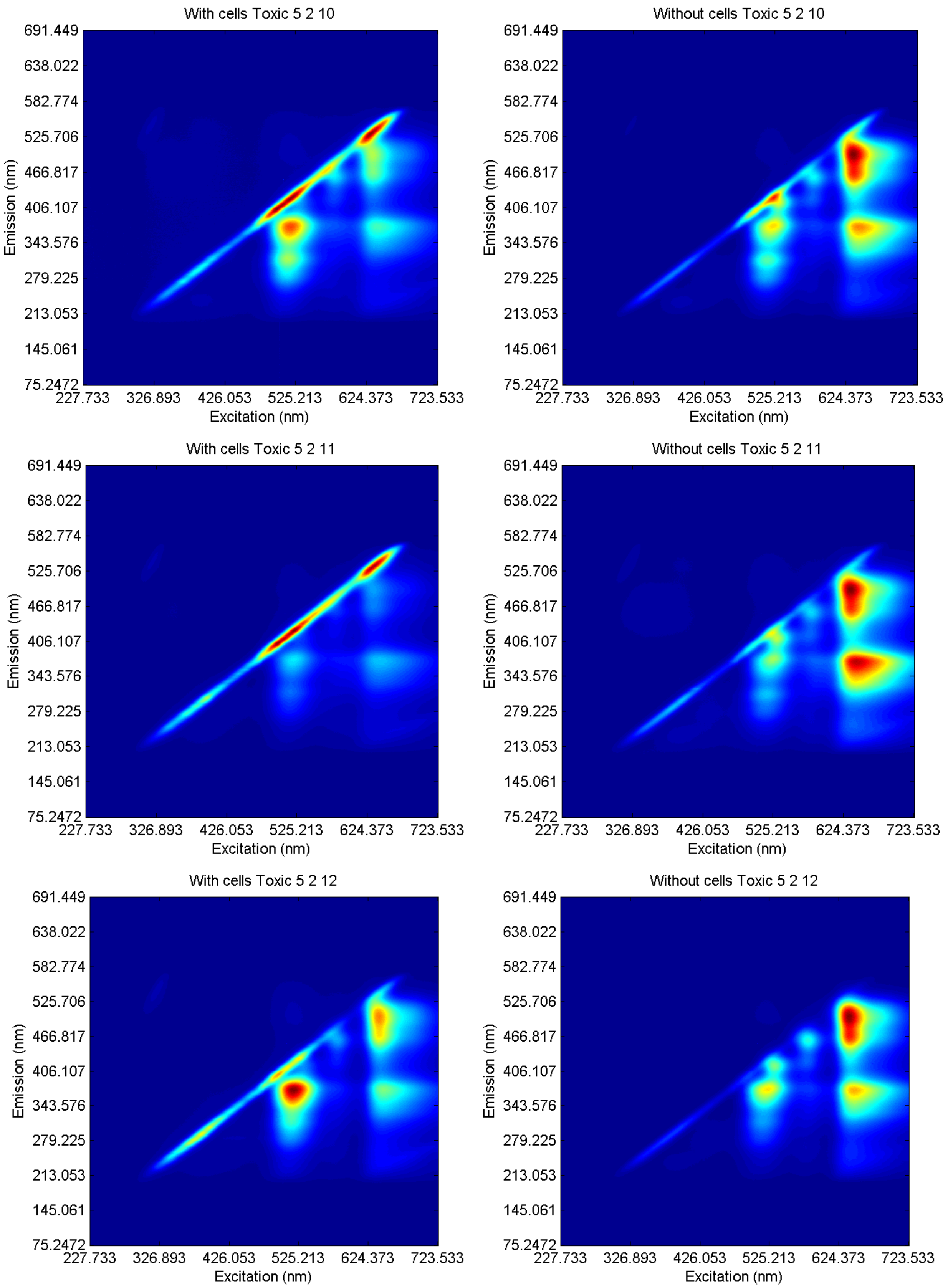
With cells Toxic 5 2 10
Without cells Toxic 5 2 10
With cells Toxic 5 2 11
Without cells Toxic 5 2 11
With cells Toxic 5 2 12
Without cells Toxic 5 2 12
Emission (nm)
691.449
638.022
582.774
525.706
466.817
406.107
343.576
279.225
213.053
145.061
75.2472
227.733
326.893
426.053
525.213
624.373
723.533
Excitation (nm)

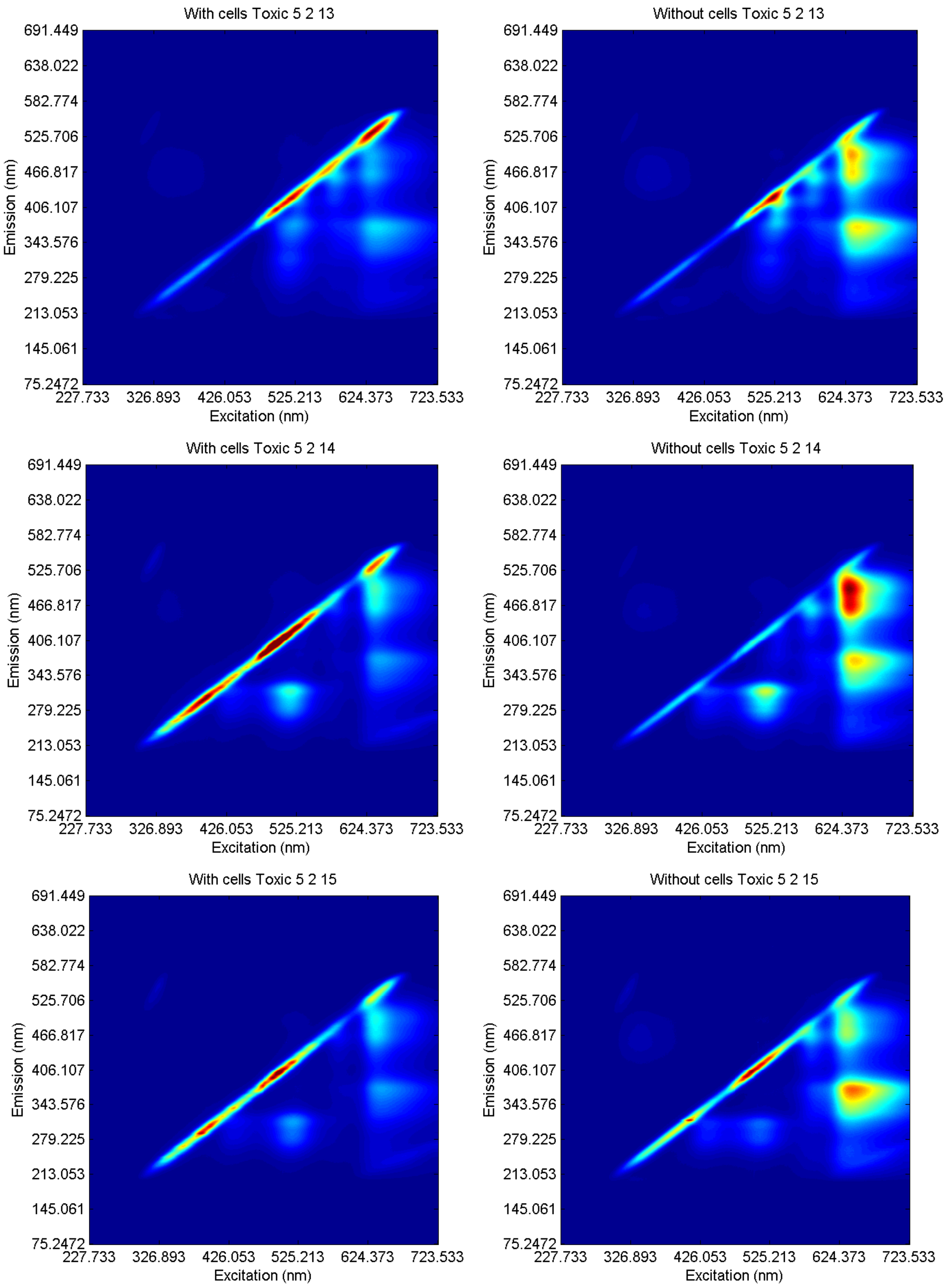
With cells Toxic 5 2 13
Without cells Toxic 5 2 13
With cells Toxic 5 2 14
Without cells Toxic 5 2 14
With cells Toxic 5 2 15
Without cells Toxic 5 2 15
Emission (nm)
Excitation (nm)
691.449
638.022
582.774
525.706
466.817
406.107
343.576
279.225
213.053
145.061
75.2472
227.733
326.893
426.053
525.213
624.373
723.533

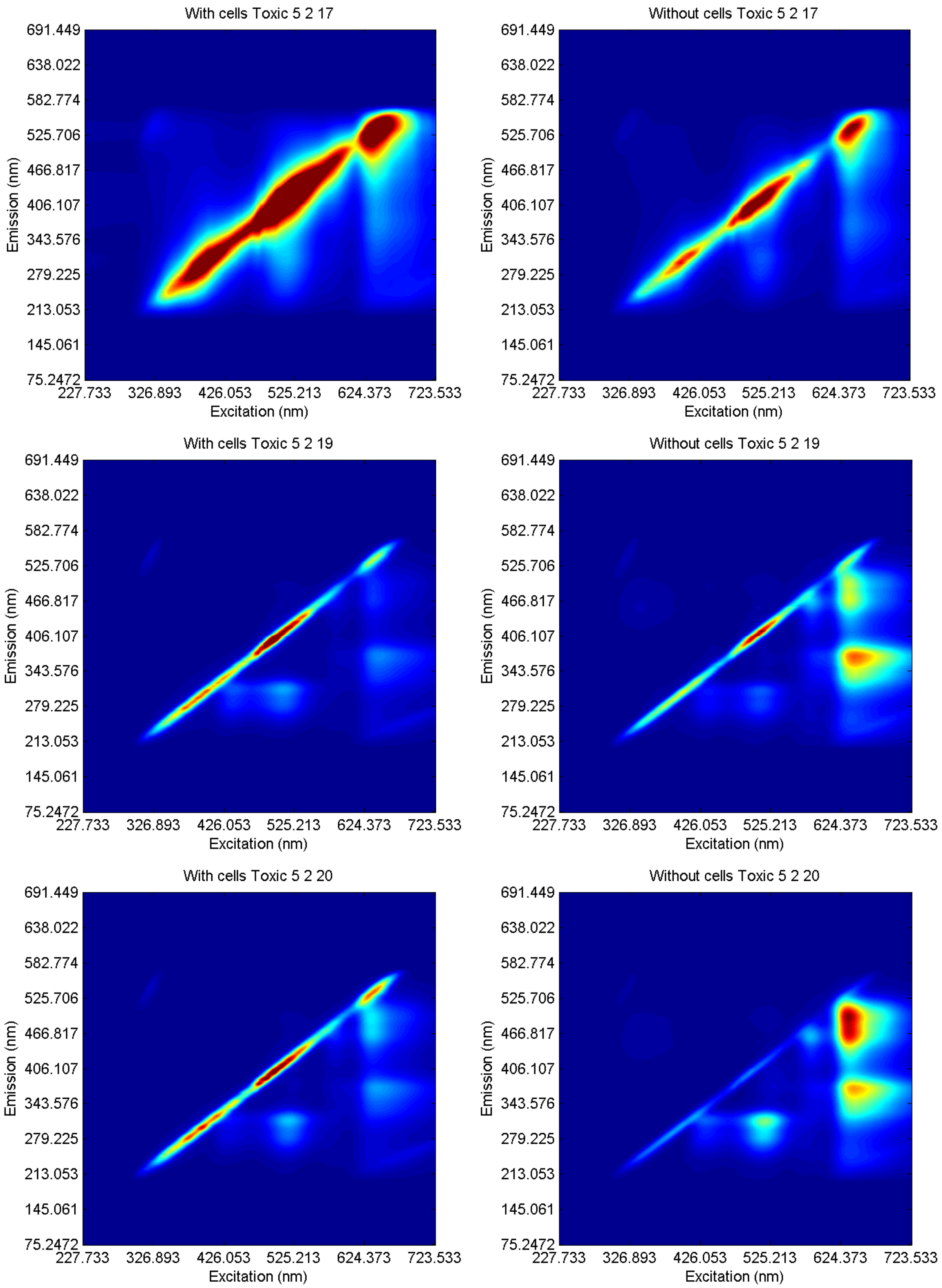

With cells Toxic 5 2 17
Without cells Toxic 5 2 17
With cells Toxic 5 2 19
Without cells Toxic 5 2 19
With cells Toxic 5 2 20
Without cells Toxic 5 2 20
Emission (nm)
691.449
638.022
582.774
525.706
466.817
406.107
343.576
279.225
213.053
145.061
75.2472
Excitation (nm)
227.733
326.893
426.053
525.213
624.373
723.533
